# Generation and Nitric Oxide Reactivity of a Cobalt(II) Superoxide Complex via Guanidine-Based Ligand Non-Innocence

**DOI:** 10.1021/jacsau.5c00418

**Published:** 2025-06-18

**Authors:** Dibya Jyoti Barman, Thomas Lohmiller, Konstantin Krause, Sagie Katz, Michael Haumann, Peter Hildebrandt, Kallol Ray

**Affiliations:** † Institut für Chemie, 9373 Humboldt-Universität zu Berlin, Brook-Taylor-Straße 2, 12489 Berlin, Germany; ‡EPR4Energy Joint Lab, §Department Spins in Energy Conversion and Quantum Information Science, Helmholtz-Zentrum Berlin für Materialien und Energie GmbH, Albert-Einstein-Straße 16, 12489 Berlin, Germany; ∥ Department of Chemistry, 26524 Technische Universität Berlin, Straße des 17. Juni 135, 10623 Berlin, Germany; ⊥ Department of Physics, 9166 Freie Universität Berlin, Arnimallee 14, 14195 Berlin, Germany

**Keywords:** bioinorganic chemistry, ligand non-innocence, peroxynitrite, oxidation state, spectroscopy

## Abstract

A novel guanidine-based NNN pincer cobalt­(II) complex, **Co1**, was investigated for dioxygen (O_2_) activation. Upon reaction with O_2_ at room temperature, a μ-hydroxo-bridged Co^II^–OH-Co^II^ complex (**Co2**) was isolated as the final oxygenated product. In-depth spectroscopic investigation revealed that the formation of **Co2** occurs via an intermediate superoxide species (**Co1–O**_**2**_^**•–**^), by taking advantage of the redox non-innocence behavior of the ligand (3,3′-(pyridine-2,6-diyl)­bis­(1-methyl-*N*-phenylimidazolidin-2-imine)), and therefore keeping the cobalt oxidation state unchanged at +2. Interestingly, the zinc analogue, **Zn1**, was also shown to yield a similar μ-hydroxo-bridged Zn^II^–OH-Zn^II^ complex (**Zn2**) as the final product under the same aerobic conditions, providing further confirmation of the ligand influence on metal oxidation and spin states during O_2_ activation. Further reactivity studies demonstrated that **Co1–O**_**2**_^**•–**^ reacts with nitric oxide (^•^NO) to form a Co^II^-peroxynitrite (**Co1–O** **NO_2_** ^**–**^ **)** intermediate, which performs an unprecedented intramolecular nitration of the phenyl moieties of the ligand at the para position.

## Graphical abstract

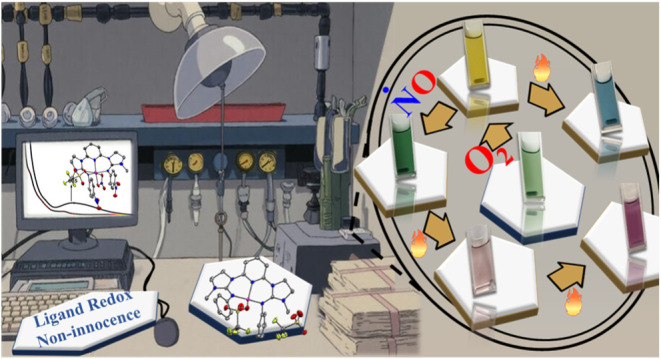

## Introduction

Peroxynitrite (^**–**^OON=O) is an incredibly reactive species that is generated by the near diffusion-controlled combination of nitric oxide (^•^NO) and the superoxide (O_2_^•–^) anion.^1,2^ It is a fascinating mediator of nitric oxide biochemistry and oxidative/nitrative stress injury.^3–9^ Metal ions in biological systems are key players in the generation and stabilization of OON=O^–^, which undergo various thermal transformation reactions like isomerization to nitrate (NO_3_^–^) or activation toward substrate oxidation/nitration reactions.^10–15^ Heme proteins have been extensively investigated in terms of their role in peroxynitrite formation and the subsequent transformation to nitrate.^5,6,15^ For example, nitric oxide dioxygenases (NOD) are known to be important enzymes that convert ^•^NO to nitrate using O_2_, potentially via peroxynitrite intermediates.^16,17^ The biological relevance of ^**–**^OON=O inspired a wide range of theoretical and experimental studies of its coordination and interaction with transition metal centers and subsequently, its reactivity. However, the formation of discrete metal-peroxynitrite complexes is a rare occurrence, but they have been suggested to form as transient intermediates from metal-NO + O_2_(g) or metal-O_2_ + ^•^NO­(g) reactions.^18–26^

Following our interest in cobalt oxidative chemistry, we note that the literature on solution chemistry of the cobalt ion with peroxynitrite is limited; no examples of isolable cobalt­(II)-peroxynitrite species with the direct use of O_2_ and ^•^NO have been described. Clarkson and Basolo first reported the chemistry of a cobalt-nitrosyl complex with O_2_, in which a transient cobalt­(III)-peroxynitrite intermediate was proposed to form the nitrite-bound product.^27^ Similarly, Co­(III)-nitrosyl complexes of 12-, 13-, and 14-membered N-tetramethylated cyclam ligands, [(12-TMC)­Co^III^(NO)]^2+^, [(13-TMC)­Co^III^(NO)]^2+^, and [(14-TMC)­Co^III^(NO)]^2+^ were reported to react with the superoxide radical and O_2_, respectively, resulting in Co­(II)-nitrite/nitrate, where the involvement of a Co­(III)-peroxynitrite intermediate was presumed.^11,21^ In addition, Mondal and co-workers suggested the formation of a transient and nonisolable Co­(II)-peroxynitrite intermediate in the reaction of a Co­(III)-peroxo species with ^•^NO.^28,29^ The formation of cobalt-peroxynitrite chemistry is also biologically relevant; cobalamins (Cbls) are known to react with ^•^NO to yield nitrosocobalamins (NOCbls), which further react with O_2_ to form nitrocobalamin through the presumable formation of peroxynitrite Co­(III) intermediate.^30–32^ The formation of cobalt-peroxynitrite is initiated in most cases upon addition of an O_2_ or ^•^NO at Co^II^ centers to yield kinetically inert low-spin terminal Co^III^ superoxide or nitrosyl moieties, which then react with ^•^NO or O_2_/O_2_^•–^/O_2_^2–^, respectively, to form Co-OONO moieties.

Herein, we report the synthesis of a novel cobalt­(II) complex, **Co1**, supported by a guanidine-based NNN pincer ligand (**L** = 3,3′-(pyridine-2,6-diyl)­bis­(1-methyl-*N*-phenylimidazolidin-2-imine)), which enables the activation of O_2_ to superoxide via ligand-based electron transfer, leading to a cobalt­(II) superoxo complex (**Co1–O**_**2**_^**•–**^). In the presence of ^•^NO, **Co1–O**_**2**_^**•–**^ forms a cobalt­(II) peroxynitrite moiety, **Co1–O**_**2**_ **NO** ^**–**^ (Scheme 1). Interestingly, the thermal decay of **Co1–O**_**2**_ **NO** ^**–**^ at room temperature leads to the nitration of phenyl groups of the ligand at the *para* position to yield a unique cobalt­(II)-nitrate species (**Co3**). Although metal-peroxynitrite-mediated nitration of phenols is known in chemistry and biology,^11–14,24,33–36^ nitration of aromatic hydrocarbons is elusive to date. The present study, therefore, opens up new ways for the nitration of aromatic hydrocarbons in the presence of ^•^NO/O_2_ by employing a ligand-based reservoir of electrons. This may offer significant improvements over the more conventional method of nitration in the presence of an HNO_3_/H_2_SO_4_ mixture, with regard to mild reaction conditions and green aspects by avoiding toxic catalysts and solvents.^37–39^

**Scheme 1:**
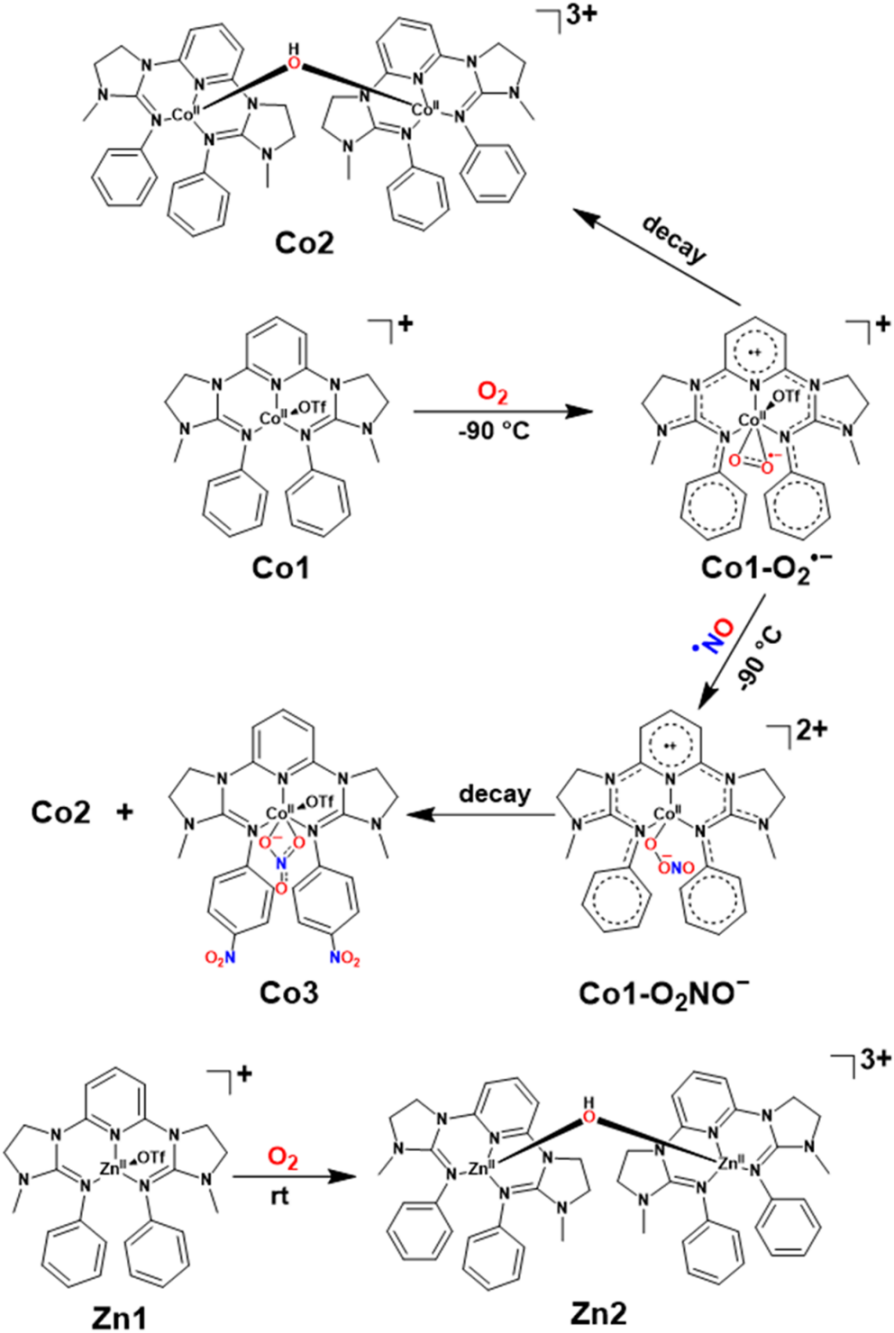
Schematic Representation of the Studied Reactions of the Cobalt and Zinc Complexes

## Results and Discussion

### Synthesis and Characterization of the Co­(II) and Zn­(II) complexes

The ligand **L** was synthesized via a Suzuki-Miyaura coupling reaction of 2,6-bromopyridine with two equivalents of the guanidine building block (**G1**, Scheme S1). ^1^H and ^13^C NMR confirm the coupling of two units of **G1** in 2 and 6 positions of the pyridine moiety (Figures S1, S2). Thereafter, complex **Co1** was prepared under an inert atmosphere by reacting **L** with an equimolar amount of cobalt­(II) triflate in acetonitrile at room temperature, giving rise to a greenish-red clear solution (see SI section 2). The zinc­(II) analogue, **Zn1**, was also synthesized by using a similar procedure employing zinc­(II) triflate. Single crystals of **Co1** were grown by diffusing diethyl ether vapors into a concentrated acetonitrile solution of the complex at −40 °C. X-ray diffraction (XRD) analysis shows that **Co1** exhibits a four-coordinate (geometry index τ_4_′ = 0.81)^40^ distorted tetrahedral cobalt center (Figure 1A). It is bound by the two imine N atoms of the **G1** units along with coordination from the pyridine N atom, forming an NNN-type pincer complex. The fourth coordination site is occupied by one of the two triflate anions in the unit cell, while the other one is unbound. The observed average Co–N bond distance is 1.961(3) Å, and the Co–O_triflate_ distance is 1.981(2) Å for the bound triflate. In ^1^H NMR, **Co1** shows paramagnetically shifted resonances, whereas **Zn1** shows a diamagnetic signal. Notably, both triflates in **Co1** exist as free anions in solution, as evident from the presence of only one peak at −71.41 ppm in ^19^F NMR measurements (Figure 1B) corresponding to unbound triflate anions.

**Figure 1:**
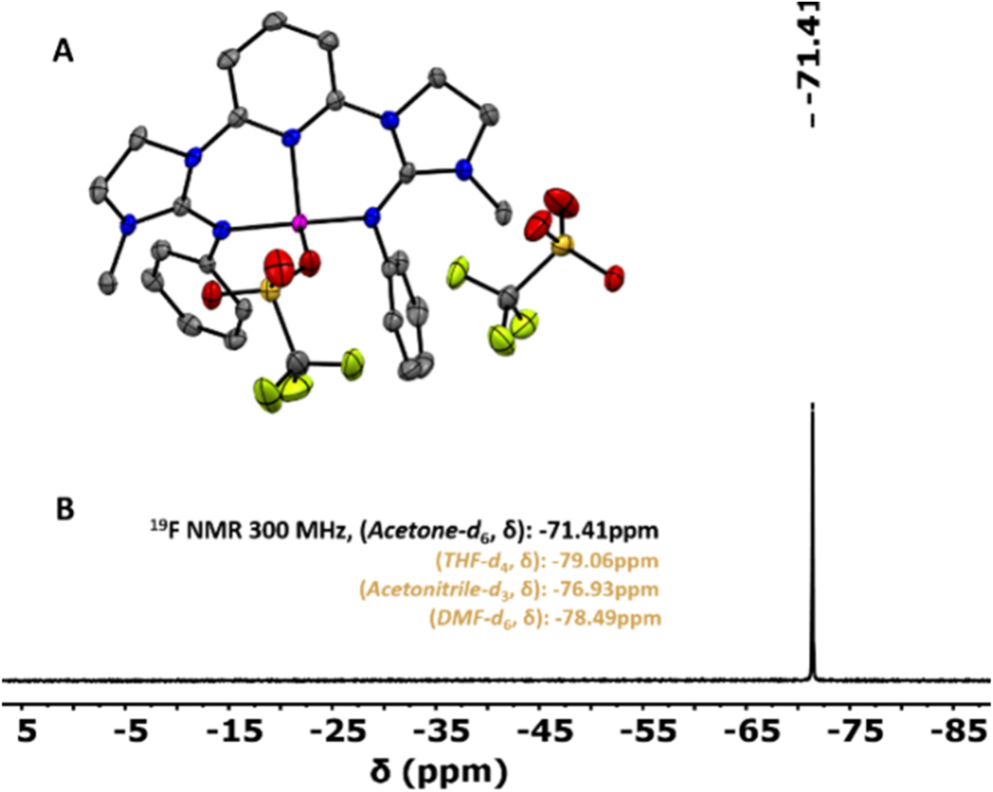
(A) XRD determined molecular structure of **Co1**, showing one bound and one unbound triflate anions per cobalt center in the solid state (one of the two molecules in the P2_1_/_C_ space group). Hydrogen atoms are omitted for clarity. Ellipsoids are drawn at the 50% probability level. (B) ^19^F NMR spectrum of **Co1** in acetone-*d*_6_.

### Cyclic Voltammetry (CV)

Cyclic voltammetry measurements were performed for both complexes **Co1** and **Zn1** in CH_2_Cl_2_ (Figure S3). Interestingly, the CV traces were found to be nearly identical in both cases; similar nonreversible waves in the region of *E*_1/2_ = −0.2 to 0.8 V vs Fc^+^/Fc were obtained, presumably referring to only ligand-based oxidation processes. The X-band electron paramagnetic resonance (EPR) spectrum of **Co1** in a frozen CH_2_Cl_2_ solution at 13 K is consistent with the presence of a high-spin S = 3/2 (Figure 2, black spectrum) cobalt­(II) ion in **Co1**. The corresponding **Zn1** complex is EPR-silent, as expected.

**Figure 2:**
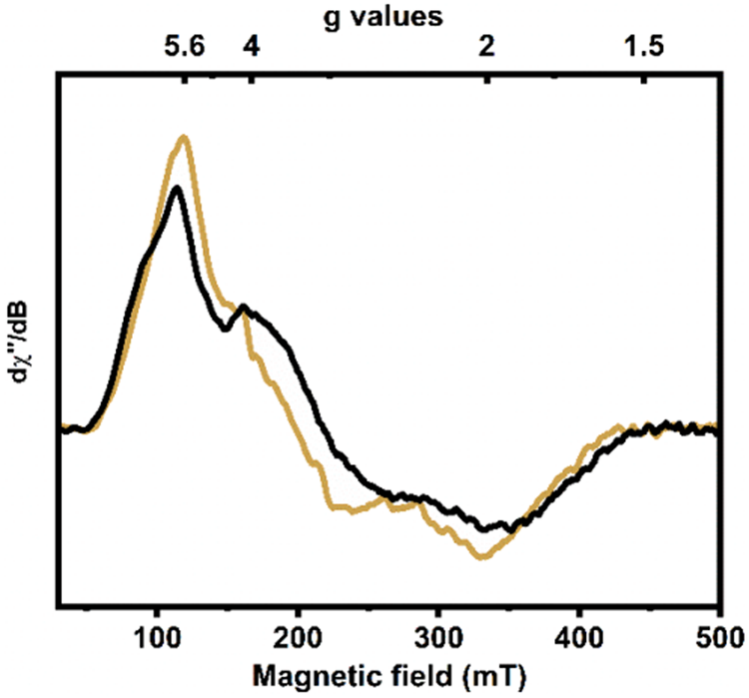
X-band EPR spectrum of **Co1** (black) and **Co1–O**_**2**_^**•–**^ (golden) in CH_2_Cl_2_ (1 mM) at 13 K. Conditions: microwave frequency 9.355 GHz; microwave power 0.016 mW; modulation amplitude 5 G.

### Oxygen Reactivity of **Co1** and **Zn1**

Bubbling dioxygen to a 1 mM solution of **Co1** in dry CH_2_Cl_2_ at −90 °C resulted in a slow but steady color change of the pale greenish solution to dark yellow (over an hour); the appearance of new absorption bands (Figure 3) was observed at 395 nm (ε = 2120 M^–1^ cm^–1^), 465 nm (ε = 960 M^–1^ cm^–1^), and 590 nm (ε = 460 M^–1^ cm^–1^). This oxygenated species, **Co1–O**_**2**_^**•–**^, which is indefinitely stable at −90 °C and below, decays immediately upon increasing the temperature to room temperature to form a blue-purple solution. Crystallization of the final decay product from a concentrated tetrahydrofuran (THF) solution afforded an EPR-silent dicobalt­(II) complex, **Co2** (Figure 4A), featuring a hydroxo-bridging unit and isolated in high yield. SQUID magnetometry revealed a comparatively weak antiferromagnetic interaction of the two high-spin cobalt­(II) centers with an exchange coupling constant (*J*) of −17.4 cm^–1^ (Figure S8). The formation of a similar hydroxo-bridged dizinc­(II) complex, **Zn2** (Figure 4B and Figures S5–S7), was obtained in high yield as a final product when **Zn1** was treated with O_2_. Both **Co2** (τ_4_′ = 0.83 and 0.77)^37^ and **Zn2** (τ_4_′ = 0.81 and 0.77)^37^ exhibit two distorted tetrahedral four-coordinate Co­(II) and Zn­(II) centers (Co–Co and Zn–Zn distances are 3.322(2) and 3.402(3) Å, respectively), with average Co–N and Zn–N bond distances of 1.971(2) and 1.984(3) Å, respectively. The average Co–O_hydroxide_ and Zn–O_hydroxide_ distances correspond to 1.900(2) and 1.909(2) Å, respectively.

**Figure 3:**
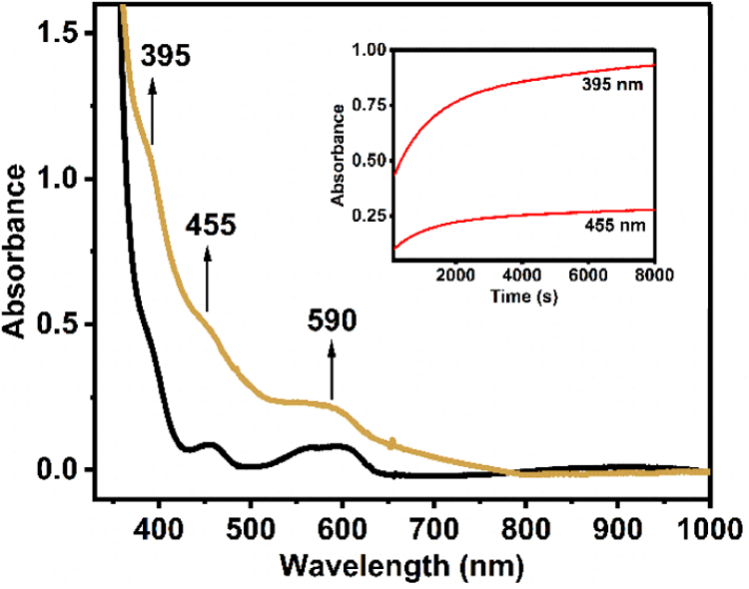
UV–vis spectral changes associated with the formation of **Co1–O**_**2**_^**•–**^ (golden) generated by bubbling O_2_ through a dry CH_2_Cl_2_ solution of **Co1** (1 mM, black) at – 90 °C. The inset shows the time trace of the development of the bands at 395 and 455 nm.

**Figure 4:**
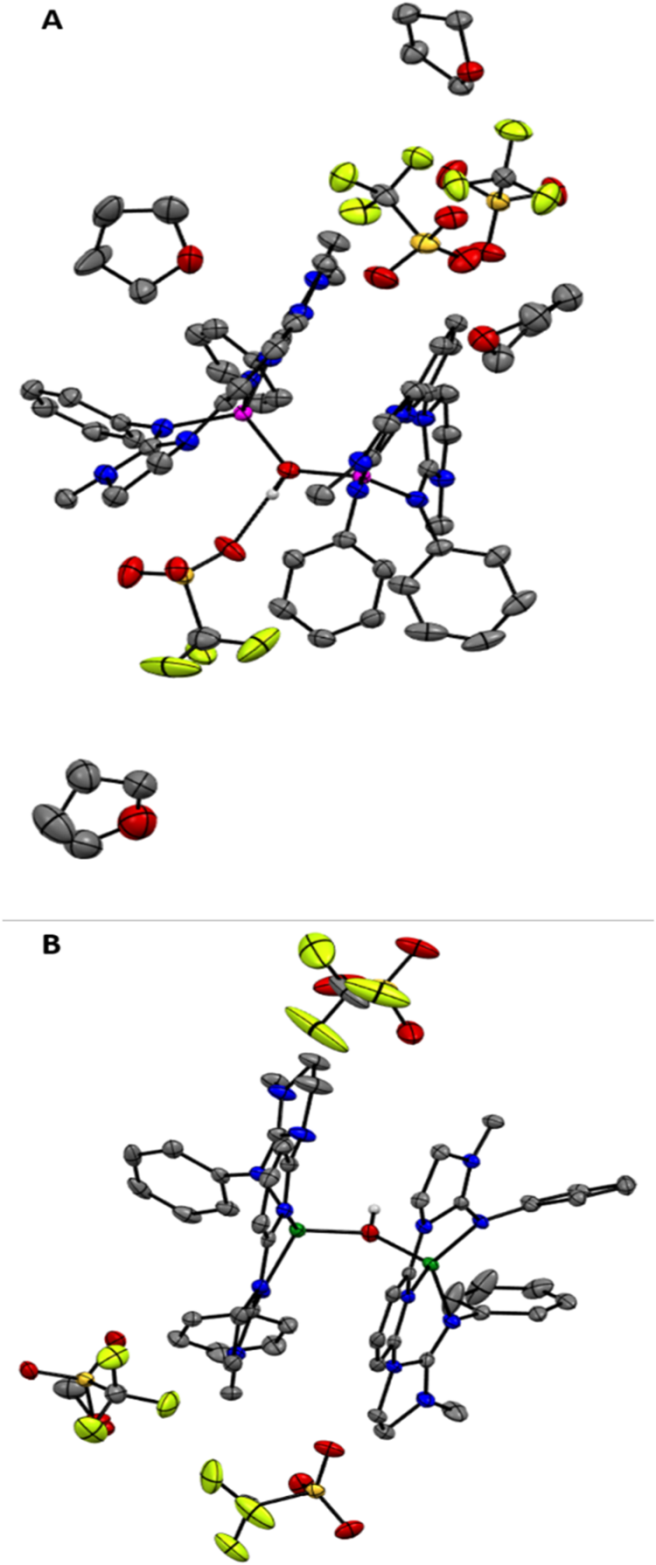
XRD determined molecular structures of (A) **Co2** with cocrystallized tetrahydrofuran solvent molecules and (B) **Zn2**. Hydrogen atoms (except for the bridging μ–OH of the metal^II^–OH-metal^II^ core) are omitted for clarity. Ellipsoids are drawn at the 50% probability level.

### Resonance Raman (rRaman) and EPR Characterizations of **Co1–O**_**2**_^**•–**^

Resonance Raman spectroscopic measurements on **Co1–O**_**2**_^**•–**^ at −90 °C using 407 nm excitation revealed the presence of a signal at 1201 cm^–1^, which upon ^18^O-labeling shifts to 1138 cm^–1^, indicating the formation of a superoxo species (Figures 5 and S9). This downshift of 63 cm^–1^ is consistent with the expected shift of 68 cm^–1^ calculated by Hooke’s law for an O–O diatomic vibration. Noticeably, the (O–O) vibrational frequency observed in this case is higher than that for previously reported cobalt superoxo species, which are known to be in the typical range of 1050–1160 cm^–1^.^41–47^ The X-band **EPR** signal of the **Co1–O**_**2**_^**•–**^ intermediate (Figure 2, golden spectrum) appears to remain mostly unaltered compared with its cobalt­(II) precursor complex, indicating that the electronic structure at the metal center remains unchanged upon dioxygen activation. As the **Co1** complex behaves electrochemically similar to the zinc analog, **Zn1** (see above), the ligand **L** is anticipated to provide the required single electron to activate dioxygen to form the superoxo intermediate, **Co1–O**_**2**_^**•–**^, at low temperature. The spins of the radical moieties **L** ^•+^ and **O**_**2**_^**•–**^ must then exhibit a strong antiferromagnetic exchange interaction. Accordingly, the EPR signal of **Co1–O**_**2**_^**•–**^, resembling that of **Co1**, is dominated by the cobalt­(II) spin. Similar strong antiferromagnetic interactions between different radical centers, dominating over the coupling to the spin of the metal, are precedent in the literature.^48–55^ Possibly, the metal ions in **Co1** and **Zn1** behave as spectators providing Lewis acidic templates for the substrate binding site, thereby helping in polarizing O_2_, while the delocalized π-system of the guanidine-based pincer framework enables charge redistribution, driving ligand-centered redox processes critical for small-molecule activation.^56^ The ligand-centered oxidation is further corroborated by the formation of a similar hydroxo-bridged zinc analogue, **Zn2**, as a final product when **Zn1** is exposed to aerobic conditions. Similar to the cobalt case, **Zn1–O**_**2**_^**•–**^ must also be involved in the reaction, which is, however, of a transient nature and could not be isolated.

**Figure 5:**
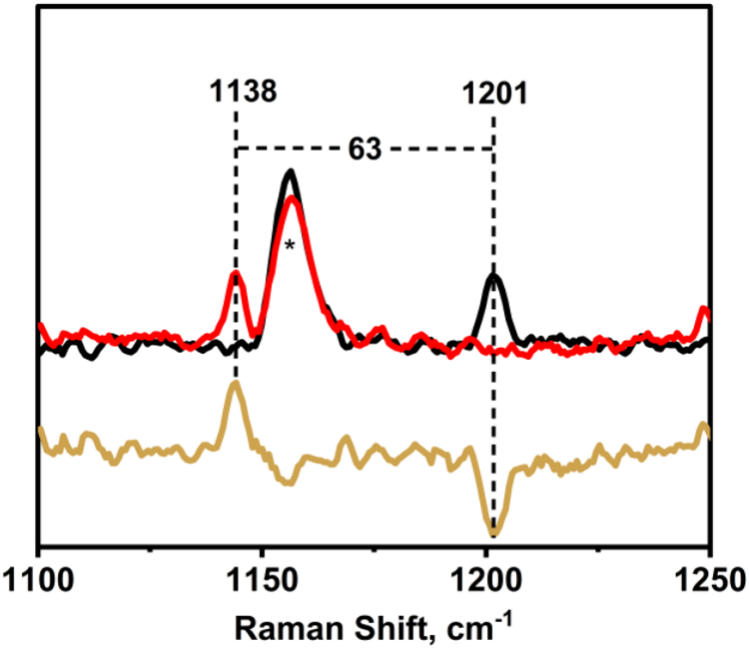
Resonance Raman (rRaman) spectra of **Co1–O**_**2**_^**•–**^ in CH_2_Cl_2_ at – 90 °C (λ_exc_ = 407 nm). Black and red traces represent the spectra of the **Co1–O**_**2**_^**•–**^ species generated with ^16^O_2_ and ^18^O_2_, respectively; the golden trace corresponds to the difference spectrum (^18^O_2_ – ^16^O_2_). (The asterisks mark solvent bands.).

### X-ray Absorption Spectroscopy (XAS)

X-ray absorption spectroscopy at the Co K-edge was performed on frozen solution samples of the complexes (10 mM in acetone) to assess the metal oxidation state and coordination environment (Figure 6). The K-edge energy of the X-ray absorption near edge structure (XANES) of **Co1** indicated a cobalt­(II) oxidation level,^41–43^ which was also implied by the similar K-edge energy for **Co1–O**_**2**_^**•–**^. In agreement with the similar EPR data for both complexes, the XANES spectra further corroborate that the ligand and not the metal has become oxidized in **Co1–O**_**2**_^**•–**^.

**Figure 6:**
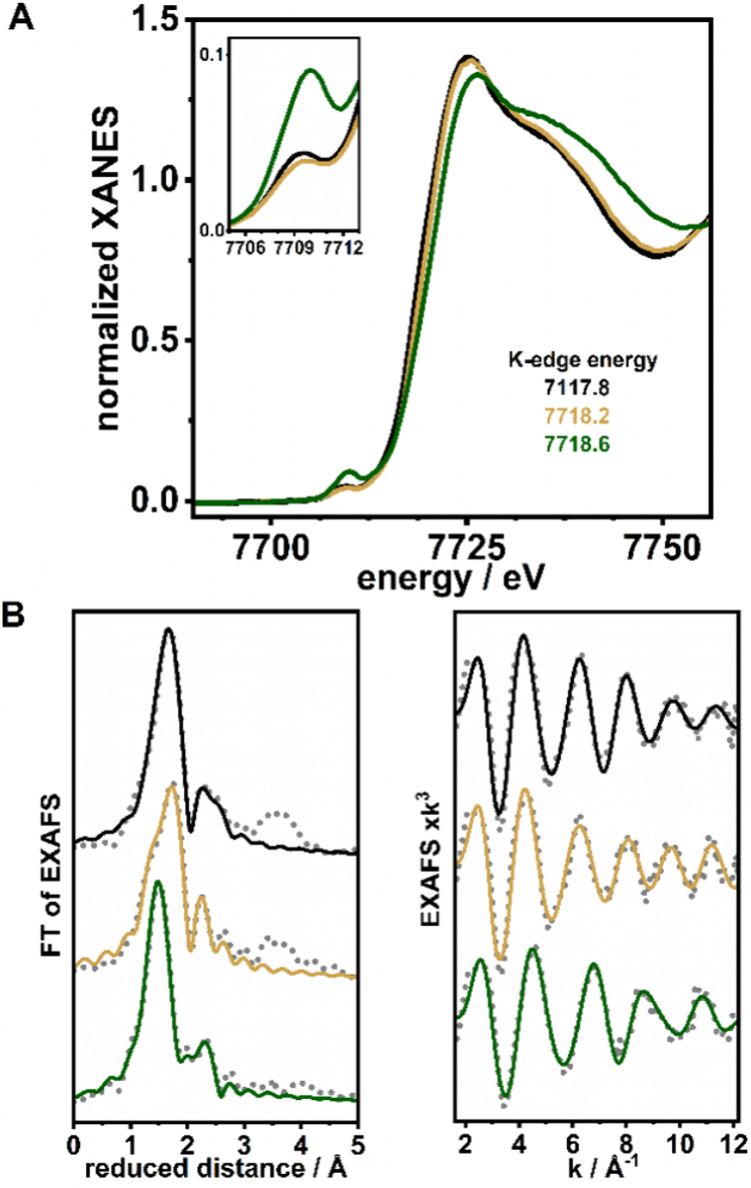
(A) Normalized Co K-edge XANES spectra for **Co1** (black), **Co1–O**_**2**_^**•–**^ (golden), and **Co1–O**_**2**_ **NO** ^**–**^ (green) in frozen acetone solutions. The inset shows an expansion of the pre-edge region. (B) Fourier transforms (left) of the respective EXAFS spectra (right; experimental data: gray dotted line; simulations: colors as for the XANES spectra). EXAFS simulation parameters are summarized in Table S3.

EXAFS spectra of the **Co1** and **Co1–O**_**2**_^**•–**^complexes are shown in Figure 6B, and their simulation parameters are summarized in Table S3. The EXAFS analysis of **Co1** suggests five ligands at the metal in an acetone solution, i.e., three Co–N_average_ (2.01 Å) from **L** and two Co–N/O (2.14 Å) bonds, possibly from triflate/solvent ligands, while the XRD structure shows only one triflate^–^ ligand at cobalt and an overall ca. 0.08 Å shorter Co–N/O bond length. The EXAFS spectrum of **Co1–O**_**2**_^**•–**^ was similarly well simulated with a five-coordinate Co center with three Co–N (1.98 Å) from **L** and two Co–N/O (2.13 Å) bonds from O_2_^•–^. The slight changes in bond lengths may be rationalized by the exchange of a solvent ligand by another species, i.e., the superoxide ligand.

### DFT Calculations

DFT calculations were performed to get insight into the nature of the superoxo intermediate **Co1–O**_**2**_^**•–**^. Geometry optimizations of a variety of starting structures (see SI section 8) yielded stable superoxo complexes only for the side-on binding mode. In contrast, the end-on binding mode always led to structures with significantly shorter O–O distances corresponding to a dioxygen unit often dissociated from the Co center. Broken-symmetry (BS) calculations for total spin states S_t_ = 3/2 (S_1_ = 2, S_2_ = 1/2) and S_t_ = 1/2 (S_1_ = 3/2, S_2_ = 1) did not converge to true BS solutions, but yielded the same structures and wave functions as the corresponding S = 3/2 and 1/2 calculations, respectively, without using the BS approach. Consistent with the experimental data, the minimum-energy structure was found for the spin state of 3/2 (Figure 7, Table S1), which is 2.0 and 3.1 kcal/mol lower in energy than those of the S = 5/2 and 1/2 structures, respectively. However, its spin density (Figure S10) as well as inspection of the frontier orbitals indicate an **LCo­(III)-O_2_^•–^** electronic configuration, with an intermediate spin (S = 1) Co­(III) center ferromagnetically coupled to the superoxo unit and no oxidation of the ligand **L**. This is in contrast to the results from CV, XANES, and EPR (which suggest antiferromagnetic interaction of the superoxo and ligand radicals). The average Co–N bond distance is 1.896 Å, the Co–O distances are 1.880 and 1.963 Å, and the O–O distance is 1.296 Å. The calculated vibrational frequency of the O–O stretching mode is 1198 cm^–1^, downshifting by 69 cm^–1^ upon ^18^O-labeling. This is in excellent agreement with the experimental frequencies and strongly supports the side-on superoxo model. In summary, the DFT approach apparently does not provide the correct description of the electronic structure of the **Co1–O**_**2**_^**•–**^ intermediate. Nevertheless, due to the exclusive stabilization of the side-on binding mode for the superoxo ligand and the agreement of experimental and calculated O–O stretching energies, we tentatively assign **Co1–O**_**2**_^**•–**^ as a side-on superoxo complex.

**Figure 7:**
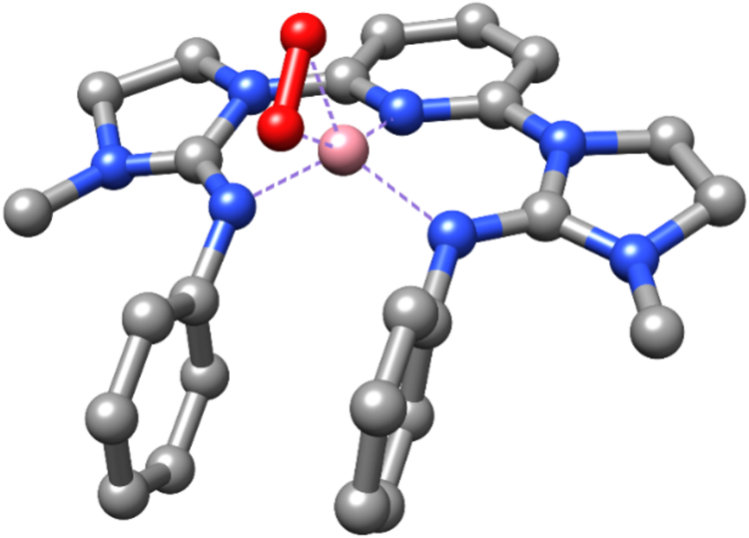
Minimum-energy (S_t_ = 3/2) DFT structure for **Co1–O**_**2**_^**•–**^. (Hydrogen atoms are omitted for the sake of clarity.).

### Reactivity with Nitric Oxide

**Co1–O**_**2**_^**•–**^ instantaneously reacts with ^•^NO at −90 °C to generate a forest green colored species, **Co1–O**_**2**_ **NO** ^**–**^, with the concomitant formation of an intense broad band at 635 nm (ε = 660 M^–1^cm^–1^) in the UV–vis absorption spectrum (Figure 8), similar to characteristic ligand-to-metal charge transfer bands of typical peroxo species.^57–62^

**Figure 8:**
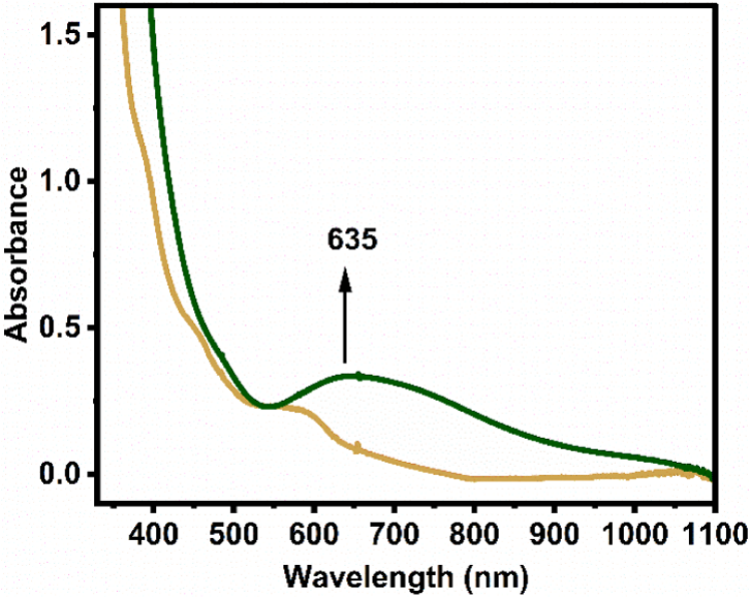
Formation of **Co1–O**_**2**_ **NO** ^**–**^ (green trace) by reaction of excess ^•^NO gas with **Co1–O**_**2**_^**•–**^ (1 mM in CH_2_Cl_2_, golden trace; cell path-length: 0.5 cm) as monitored by UV–vis spectroscopy at – 90 °C.

In the rRaman spectra of **Co1–O**_**2**_ **NO** ^**–**^ (Figure 9), the O–O stretch of **Co1–O**_**2**_^**•–**^ at 1201 cm^–1^ completely disappeared and two new ^16/18^O isotope sensitive vibrational modes were observed at 808 and 1598 cm^–1^, which upon ^18^O-labeling shift to 773 and 1595 cm^–1^, respectively. These bands are tentatively assigned to CoO-ONO and CoO_2_N=O vibrational modes, particularly in view of the similar isotopic shift pattern as in the literature.^14,63^ X-band EPR measurement (13 K) of the solution of **Co1–O**_**2**_ **NO** ^**–**^ frozen in liquid N_2_ right after the addition of ^•^NO to **Co1–O**_**2**_^**•–**^ at −90 °C shows that the S = 3/2 signal from high-spin cobalt­(II) in **Co1–O**_**2**_^**•–**^ vanishes entirely, resulting in the formation of an EPR-silent species (Figure 11A). This observation can be ascribed to spin coupling between the high-spin cobalt­(II) core and the ligand radical **L** ^•+^, resulting in an integer spin (S = 1 or 2) for **Co1–O**_**2**_ **NO** ^**–**^. A slightly higher K-edge energy (Figure 6A, green) was observed for **Co1–O**_**2**_ **NO** ^**–**^, which may suggest the formation of cobalt­(III) in part of the sample. However, the largely increased pre-edge amplitude and the broader rising edge of **Co1–O**_**2**_ **NO** ^**–**^ rather were indicative of a diminished coordination symmetry at the metal site, so that we assign Co­(II) also to **Co1–O**_**2**_ **NO** ^**–**^. The EXAFS spectrum of **Co1–O**_**2**_ **NO** ^**–**^ (Figure 6B, green) revealed only four ligands at the metal (i.e., three Co–N and one Co–N/O bonds), furthermore resulting in overall almost 0.1 Å shorter bond lengths, with the shortest bond at ca. 1.8–1.9 Å. In addition, the simulation suggested a further light atom in the second coordination sphere at a distance of ca. 2.45–2.70 Å to the metal, which was seemingly absent in the precursor complexes **Co1** and **Co1–O**_**2**_^**•–**^. This atom may stem from a diatomic (or higher atomic) N/O-containing ligand bound to the cobalt center, such as peroxynitrite (^**–**^O_2_NO). Binding of this ligand seems to cause the loss of the fifth ligand from the metal.

**Figure 9:**
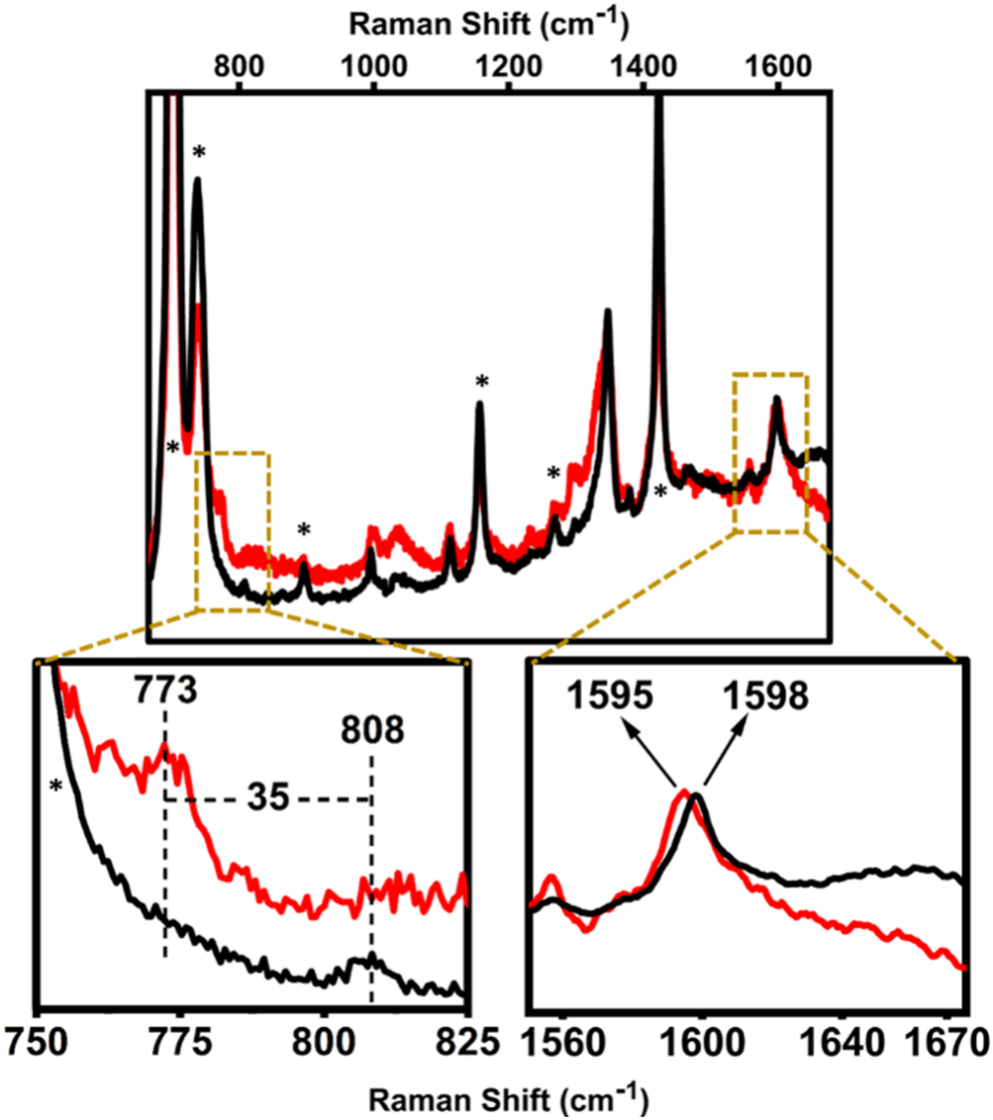
rRaman spectra of **Co1–O**_**2**_ **NO** ^**–**^ in CH_2_Cl_2_ at – 90 °C (λ_exc_ = 407 nm). Black and red spectra refer to **Co1–O**_**2**_ **NO** ^**–**^ generated from the reaction of ^•^NO with **Co1-** ^**16**^ **O**_**2**_^**•–**^ and **Co1-** ^**18**^ **O**_**2**_^**•–**^, respectively. (The asterisks mark solvent bands.).

Although **Co1–O**_**2**_ **NO** ^**–**^ is stable at −90 °C for more than an hour, it undergoes a thermal decay at higher temperatures, presumably by O–O bond homolysis to form L^•+^Co­(II)-oxyl and ^•^NO_2_ radical species, indicated by X-band EPR measurements on a reaction aliquot sample taken immediately after the reaction mixture has reached 0 °C from −90 °C during the process of warming up. The EPR-silent solution of **Co1–O**_**2**_ **NO** ^**–**^ transforms (Figure 11A,B) to a solution that exhibits an S = 3/2 signal corresponding to L^•+^Co­(II)-oxyl (10% yield based on Co) and a radical-based signal at *g* = 2 with dominant ^14^N-hyperfine splitting. The signal shape and turning points are characteristics of the ^•^NO_2_ radical as previously reported,^64–67^ which are confirmed by the ***g*** = [2.0043, 1.9901, 2.0008] and hyperfine (***A***) [142, 134, 188] MHz tensor components obtained from spectral simulations (Figure S11). The formation of ^•^NO_2_ is further confirmed by the appearance of a reddish-brown gas in the reaction vessel upon reaching room temperature (Figures S12–S13). The signal corresponding to the ^•^NO_2_ radical eventually disappears after reaching room temperature (Figure 11B,C), and the S = 3/2 cobalt­(II) signal persists with approximately 80% depleted intensity compared to the starting **Co1** complex. Extracting the final decay product with tetrahydrofuran revealed the formation of two different cobalt complexes, **Co2** and **Co3**, in 62 and 21% yields, respectively, with respect to **Co1**. The different solubilities of **Co3** and **Co2** in THF enabled their separation, with **Co3** subsequently being isolated by recrystallization of the remaining crude product from acetonitrile. The crystal structure of **Co3** (Figure 10) exhibits a six-coordinate cobalt­(II) center with two anions, one triflate and one nitrate, bound to it. The average Co–N distance is 2.084(14) Å, and Co–O_triflate_ is 2.189(10) Å. Interestingly, the generation of **Co3** is accompanied by concomitant nitration of the two phenyl rings in the ligand backbone, selectively at the *para* positions. When the thermal decay of **Co1–O**_**2**_ **NO** ^**–**^ was performed in the presence of an external substrate like anisole, both *ortho* and *para*-nitro anisole were obtained in 37.2% yield each, with respect to the peroxynitrate species (see SI section 2 and Figure S14).

**Figure 10:**
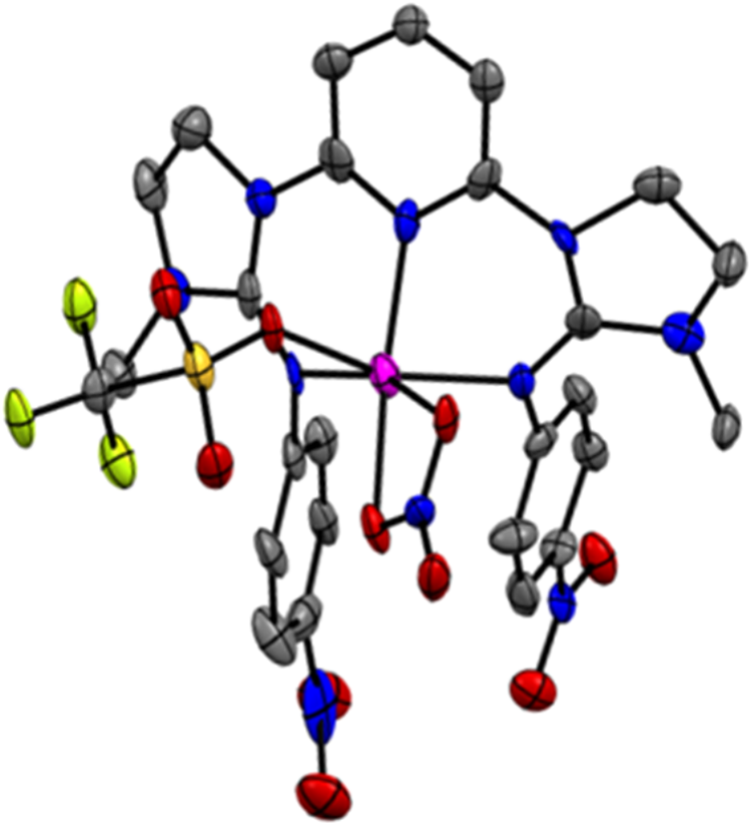
XRD determined molecular structure of **Co3**. (Hydrogen atoms are omitted for clarity. Ellipsoids are drawn at the 25% probability level.).

**Figure 11:**
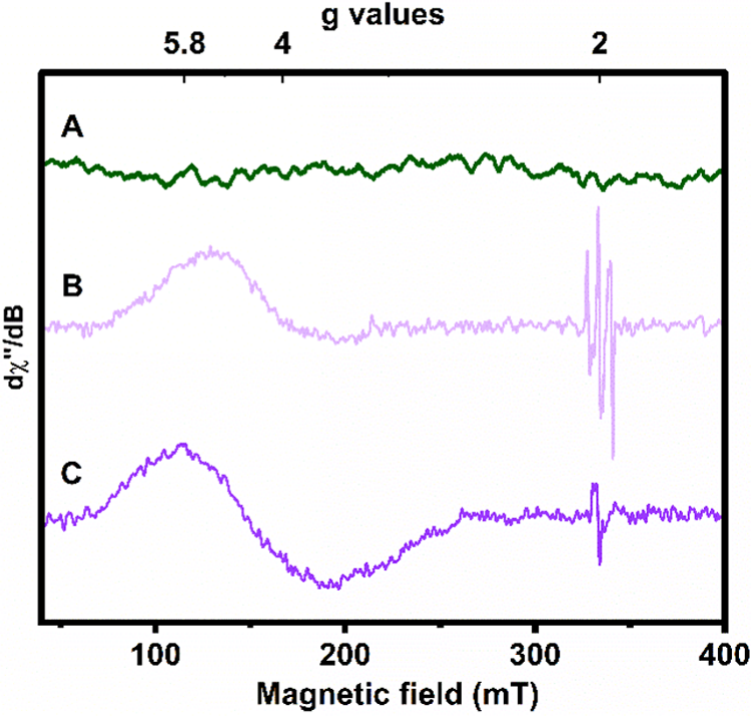
Monitoring of the thermal decomposition of **Co1–O**_**2**_ **NO** in CH_2_Cl_2_ (1 mM) by X-band EPR spectroscopy: (A) initial spectra corresponding to **Co1–O**_**2**_ **NO** at – 90 °C; (B) transient formation of L^•+^Co­(II)-oxyl (10% yield) and ^•^NO_2_ as observed from a reaction mixture aliquot taken immediately after warming to 0 °C; and (C) the final formation of **Co3** (20% yield) at room temperature. Conditions: microwave frequency 9.355 GHz, microwave power 0.016 mW, modulation amplitude 5 G, and temperature 13 K.

Thus, L^•+^Co­(II)-oxyl and ^•^NO_2_, formed upon O–O bond homolysis of **Co1–O**_**2**_ **NO** ^**–**^, may either rearrange to form a nitrate anion, or the ^•^NO_2_ radical in the presence of L^•+^Co­(II)-oxyl may be responsible for the nitration of the phenyl rings in the *para* positions of the ligand. The plausible reaction sequence for the generation of **Co3** and **Co2** from the thermal decay of **Co1–O**_**2**_ **NO** ^**–**^ is shown in Scheme 2. Consistent with the proposed mechanism, the decay of **Co1–O**_**2**_ **NO** ^**–**^ is found to be dependent on cobalt concentration (Figure S12), thereby suggesting an intermolecular mechanism. In addition, dioxygen is detected by gas chromatography (Figure S13). Notably, thermal and photochemical nitration of aromatic hydrocarbons with ^•^NO_2_ has been reported as an alternative to the usual electrophilic substitution pathway for aromatic nitration reactions involving nitrosonium cations (NO_2_^+^).^68,69^ The present study establishes a plausible synergistic role of metal-oxo and ^•^NO_2_, obtained from the decay of metal-peroxynitrites, in mediating aromatic nitration reactions under mild conditions in a greener way by avoiding toxic catalysts and solvents. These aspects will be investigated in detail in future studies.

**Scheme 2:**
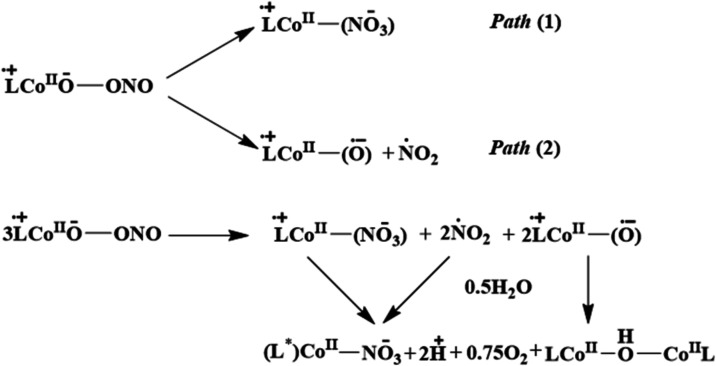
Proposed Reaction Scheme for the Thermal Decomposition of **Co1–O**_**2**_ **NO** to the (L*)­Co^II^ Moiety in **Co3** and the L-Co^II^–OH-Co^II^L Unit in **Co2** ^a^

a (L*) represents the double nitration product of the ligand, **L**. L^•+^Co­(II)-oxyl presumably acts as the H-atom abstraction agent during the nitration reaction.

## Conclusions

Herein, we demonstrated activation of O_2_ via a ligand-based redox process to generate an unusual Co­(II) superoxo species. The superoxo complex was characterized by EPR, XAS, and rRaman spectroscopic techniques to validate the proposed ligand-based reactivity pathway. This complex can react with ^•^NO to form a Co peroxynitrite species, which performs an unprecedented intermolecular aromatic nitration reaction. The observed ligand-based electron transfer to NO/O_2_ may be related to that in biological systems proposed to utilize a similar cobalt-peroxynitrite intermediate.^70^ This work represents an important step forward in leveraging ligand-based redox for inexpensive and benign aromatic nitration reactions with cobalt.

## Supplementary Material



## Notes

Synthetic and spectroscopic methods, NMR, CV, and rRaman data, SQUID data and simulation, DFT computations, EPR simulation, EXAFS table, crystallographic information (PDF)
