# Supplementary material for: Generation and Nitric Oxide Reactivity of a Cobalt(II) Superoxide Complex via Guanidine-Based Ligand Non-Innocence
Source: JACS Au. 2025 Jun 18;5(7):3240–8. doi: 10.1021/jacsau.5c00418 (PMC12308394; doi:10.1021/jacsau.5c00418)
Supplement: Supplementary file 1 [file au5c00418_si_001.pdf]

Supporting Information

Generation and Nitric Oxide Reactivity of a Cobalt(II) Superoxo Complex via Ligand-Based Redox Non-Innocence

Dibya Jyoti Barman[†], Thomas Lohmiller[†‡], Konstantin Krause[†], Sagie Katz[§], Michael Haumann[#], Peter Hildebrandt[§] and Kallol Ray[†]*

[†]Institut für Chemie, Humboldt-Universität zu Berlin, Brook-Taylor-Straße 2, 12489 Berlin, Germany
[‡]EPR4Energy Joint Lab, Department Spins in Energy Conversion and Quantum Information Science; Helmholtz-Zentrum Berlin für Materialien und Energie GmbH, Albert-Einstein-Straße 16, 12489 Berlin, Germany
[§]Department of Chemistry, Technische Universität Berlin, Straße des 17. Juni 135, 10623 Berlin, Germany
[#]Department of Physics, Freie Universität Berlin, Arnimallee 14, 14195 Berlin, Germany

Table of Contents

1 General Procedures: ... 3
1.1 Chemicals: ... 3
1.2 Instrumentation: ... 3
1.3 DFT computations: ... 5
2 Synthesis of the Ligand and metal complexes: ... 6
2.1 Synthesis of Ligand (L): ... 6
2.2 Synthesis of Co1: ... 6
2.3 Synthesis of Zn1: ... 6
2.4 Synthesis of Co2: ... 6
2.5 Synthesis of Zn2: ... 7
2.6 Synthesis of Co3: ... 7
2.7 External substrate (anisole) nitration with Co1-O_2NO^-: ... 7
3 NMR spectra of L: ... 8
4 Cyclic Voltammetry: ... 9
5 NMR spectra of Zn2: ... 10
6 SQUID magnetometry of Co2: ... 12
7 rRaman spectra of Co1-$O_2^{\bullet-}$: ... 13
8 DFT computations for Co1-$O_2^{\bullet-}$: ... 14
9 EPR spectrum of the observed $^{\bullet}NO_2$ radical: ... 16
10 Peroxynitrite decay kinetic studies: ... 17
11 Dioxygen and $^{\bullet}NO_2$ detection by GC-Gas: ... 18
12 External Substrate nitration reactivity: ... 19
13 Table S2. XRD determined selected bond lengths. ... 20
14 Table S3. EXAFS simulation parameters:[a] ... 21
15 Table S4. Crystallographic data: ... 23
16 References: ... 24

1 General Procedures:

1.1 Chemicals:

All chemicals used in this study were procured from commercial suppliers, ABCR, ACROS, SIGMA-ALDRICH, TCI, and Alfa Aesar, and were utilized without further purification unless explicitly stated otherwise. The anhydrous solvents, specifically acetonitrile, acetone, diethyl ether was acquired from CARL-ROTH GmbH, marketed under the trade name ROTIDRY (purity >99.5%, with <50 ppm H_2O). Prior to usage, these solvents were degassed and subsequently stored over 4 Å activated molecular sieves in a nitrogen atmosphere.

Deuterated solvents were sourced from EURISOTOP. The preparation and manipulation of air- or water-sensitive compounds were conducted under an inert atmosphere (nitrogen or argon) utilizing either Schlenk techniques or a glovebox (GS MEGA) obtained from GS-GLOVEBOX Systemtechnik GmbH, which was maintained under a nitrogen environment. Nitrogen and argon gases of quality 5.0 were employed for these procedures and were obtained from AIR LIQUIDE.

Building block, **G1** is synthesized according to the literature reported procedure[1] where N-methylethylenediamine was used instead of ethylenediamine.

1.2 Instrumentation:

^{1}H and ^{19}F NMR spectra were acquired using a Bruker DPX 400 spectrometer, with chemical shifts expressed in parts per million (ppm) and referenced to the reported residual solvent resonances[2].

Elemental analyses were performed using a HEKAtech EURO EA 3000 analyzer.

Cyclic voltammetry experiments were performed using a Metrohm Autolab PGSTAT 204 potentiostat/Galvanostat. The experiments were done in dichloromethane with 0.10 M tetrabutylammonium hexafluorophosphate as the supporting electrolyte. A three-component electrochemical cell was used, which included a platinum working electrode, a non-aqueous pseudo-reference electrode (Ag/AgNO3), and a glassy carbon auxiliary electrode for cyclic voltammetry. All measurements were referenced against the ferrocene/ferrocenium couple.

UV-vis spectra were collected on a diode array spectrometer featuring a Unisoku Scientific Instruments cryostat. Gas addition was done either via syringe injection or using Schlenck technique into a septum-sealed 0.5 cm quartz Schlenck cuvette.

Resonance Raman spectra were recorded using the 407 nm line of a Kr^+-Laser (Coherent) and a Horiba Jobin-Yvon LabRAM HR800 confocal Raman spectrometer. The set up was connected to a Bruker cryostat and measurements were carried out at −90 °C

Electron Paramagnetic Resonance (EPR) spectra were recorded using a Bruker EMXplus instrument, operating at a frequency of approximately 9.35 GHz in perpendicular polarization mode and using a microwave power of 0.016 mW and a modulation amplitude of 5 G, on frozen solutions at an average temperature of 13 K, facilitated by a cryogen-free closed cycle helium recirculating cooling system from ColdEdge.

Spectral simulations for the $^{\bullet}NO_2$ radical were performed employing the spin Hamiltonian

$$\hat{H} = \hat{H}_{\text{Zeeman}} + \hat{H}_{\text{HFI}} = \mu_B \boldsymbol{B_0} \boldsymbol{g} \boldsymbol{\hat{S}} + \boldsymbol{\hat{S}} \boldsymbol{A} \boldsymbol{\hat{I}}, \quad \text{(eq. S1)}$$

where the first two terms account for the Zeeman and hyperfine interaction (HFI), respectively. $\boldsymbol{\hat{S}}$ and $\boldsymbol{\hat{I}}$ are the electron and ^{14}N nuclear spin operators, respectively, and $\boldsymbol{g}$ and $\boldsymbol{A}$ represent the $\boldsymbol{g}$ and ^{14}N HFI tensors, respectively.

SQUID magnetometry: Dc magnetic measurements were carried out with a QuantumDesign MPMS3 SQUID magnetometer in VSM mode on a powder sample of **Co2**. The sample was pressed and sealed under inert atmosphere into a VSM powder capsule and then mounted on a brass sample holder. Both the capsules were dried in a Schlenk flask under vacuum at 110 °C for several days. The sample was cooled down to 2 K at zero field, before recording the magnetic moment from 2 to 300 K at a constant (dc) magnetic field of 0.1 T, followed by variable-field variable-temperature (VFVT) magnetization measurements from 2 to 60 K at dc fields of 1, 4 and 7 T. A diamagnetic background correction to the raw data was applied by subtracting the magnetic moments of an empty capsule sealed with a piece of Teflon tape using the same measurement sequence as for the sample. The data were then corrected for diamagnetic contributions from the compound **Co2** using tabulated Pascal constants.[3]

The molar data were modelled using the *EasySpin* toolbox,[4-5] employing the spin Hamiltonian

$$\hat{H} = \hat{H}_{\text{Zeeman}} + \hat{H}_{\text{ZFS}} + \hat{H}_{\text{HDvV}} = \sum_{i=1}^{2} \mu_B \boldsymbol{B_0} \boldsymbol{g} \boldsymbol{\hat{S}_i} + \sum_{i=1}^{2} \boldsymbol{\hat{S}_i} \boldsymbol{D} \boldsymbol{\hat{S}_i} - 2J \boldsymbol{\hat{S}_1} \boldsymbol{\hat{S}_2}, \quad \text{(eq. S2)}$$

where the first two terms account for the Zeeman interaction and zero-field splitting (ZFS), respectively, at the cobalt(II) centres and the third term for the exchange interaction between the cobalt(II) sites using the Heisenberg-Dirac-van Vleck approach. $\boldsymbol{\hat{S}_i}$ (i = 1,2) are the electron spin operators of the cobalt(II) centres, $\boldsymbol{g}$ and $\boldsymbol{D}$ represent the on-site $\boldsymbol{g}$ and ZFS tensors, respectively, and J is the isotropic exchange coupling constant.

X-Ray Diffraction data to elucidate the crystal structures was collected at a temperature of 100 K utilizing a Bruker D8 VENTURE diffractometer equipped with a PHOTON III detector system. This instrumentation features a micro-focus X-ray tube with a Mo-target, with a wavelength of 0.71073 Å. The data reduction and integration processes were conducted using the Bruker APEX4 software package. Furthermore, the collected data were scaled and corrected for absorption effects employing the multi-scan procedure implemented in SADABS. The structure was subsequently solved employing the dual method provided by SHELXT2 and further refined through a full-matrix least-squares procedure using the OLEX2 software package.

X-ray absorption spectroscopy (XAS) at the Co K-edge was conducted at beamline KMC-3 of the BESSY-II synchrotron (Helmholtz Zentrum Berlin, Germany). The experimental setup featured (e.g.) a Si[111] double-crystal monochromator, a 13-element energy-resolving Si-drift detector (RaySpec) for X-ray fluorescence monitoring, and DXP-XMAP pulse-processing electronics (XIA). Solution samples of the Co complexes (10 mM in acetone) were maintained at 20 K in a liquid-helium cryostat (Oxford). Calibration of the energy axis of the monochromator was achieved (accuracy ±0.1 eV) utilizing the peak at 7709 eV in the first derivative of the absorption K-edge spectrum of a Co metal foil as a reference point. The beam had a spot size on the sample of approximately 1.5 x 3.0 mm^2 (vertical x horizontal) determined by a focusing mirror and slits. X-ray fluorescence spectra were acquired using a continuous

scan mode of the monochromator, with each scan taking about 8 minutes. Up to five scans (1-2 per sample spot) were averaged. The XAS data were processed (dead-time correction, background subtraction, normalization) resulting in normalized XANES and EXAFS spectra. The k^3-weighted EXAFS spectra were simulated using in-house software and phase functions derived from the FEFF9 code.

Gas chromatography (GC): Shimadzu GC-2014 gas chromatograph was used for gas detection with a thermal conductivity detector and a Resteks ShinCarbon packed column ST 80/100 (2 m, 1/8" outer diameter, 2 mm inner diameter). The injector temperature was set to 200 °C, the detector temperature set to 300 °C and the gases were separated according to a temperature-time program on the column. Gases from the headspace were separated at the early stages of the measurement at an oven temperature of 40 °C.

1.3 DFT computations:

DFT calculations (spin-unrestricted) were carried out using ORCA 6.0.1.[6-9] Relativistic effects were considered using the zeroth-order regular approximation (ZORA),[10-12] employing the specially adapted segmented all-electron relativistically recontracted basis set ZORA-def2-TZVP[13]. The conductor-like polarizable continuum model (C-PCM)[14] with the solvent properties of DCM was used. Tight self-consistent field (TightSCF) convergence criteria were employed. The atom-pairwise dispersion correction with Becke-Johnson damping (D3BJ)[15] was applied to the DFT energy. The hybrid functional B3LYP[16-18] with the resolution of identity (RI) approximation to the Coulomb exchange and the chain-of-spheres approximation to exact exchange (RIJCOSX)[19-20] along with the decontracted auxiliary basis set SARC/J[21] was employed in geometry optimizations. Frequency calculations were performed using the density functional BP86[16, 22] with the RI approximation[23] together with the SARC/J auxiliary Coulomb fitting basis set.

Molecular images were generated using UCSF Chimera.[24]

2 Synthesis of the Ligand and metal complexes:

2.1 Synthesis of Ligand (L):

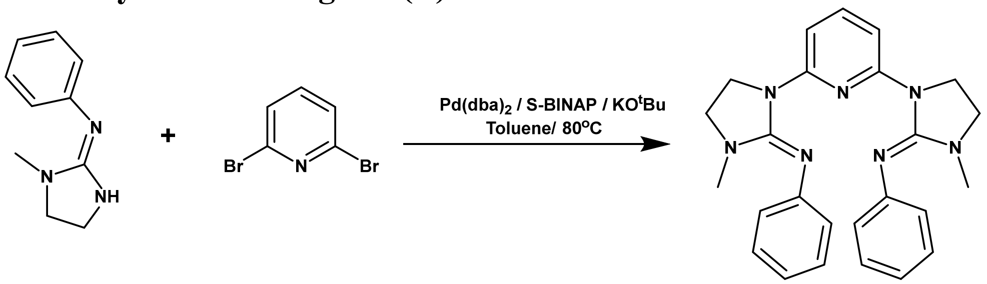

Scheme S1. Schematic diagram of ligand synthesis.

In an oven-dry Schlenk flask building block, **G1** (386 mg, 2.2 mmol), 2,6-dibromopyridine (237 mg, 1 mmol), and KOtBu (337 mg, 3 mmol) were added to a pre-mixed toluene solution containing [Pd(dba)$_2$] (115 mg, 0.2 mmol) and (S)-BINAP (310 mg, 0.5 mmol). The mixture was stirred for 30 minutes at room temperature and then was heated at 80 °C for 24 hours. Upon cooling, 100 mL of deionized water was added, followed by extraction twice with dichloromethane. The organic layers were dried, filtered, and evaporated to yield a red oil. 50 ml of methanol was added and kept at 5 °C overnight, during which precipitation began to accumulate. The solids were isolated by filtration, washed, and dried, resulting in beige solid.
Yield: 390mg (92 %). Anal. Calc. for $C_{25}H_{27}N_7$: C, 70.56; H, 6.40; N, 23.04. Found: C, 70.54; H, 6.39; N, 23.01.

2.2 Synthesis of Co1:

In a 15 mL vial Co(CF$_3$SO$_3$)$_2$ (350 mg, 0.99 mmol) was added as solid to a solution of **L** (430 mg, 1.0 mmol) in CH$_3$CN (3.0 mL) under inert atmosphere. After stirring the reaction mixture at room temperature for 24 hours, it was filtered with a 0.43 µm PTFE syringe filter. The filtrate was dropwise added to a 20 mL vial full of diethyl ether to yield voluminous pale greenish-red precipitation, and the supernatant solution was then decanted. The remaining solid was washed twice with diethyl ether (5 mL) and vacuum-dried afterwards.
Yield: 0.76 g (98 %). Anal. Calc. for $C_{27}H_{27}CoF_6N_7O_6S_2$: C, 41.44; H, 3.48; N, 12.53; S, 8.19. Found: C, 41.58; H, 3.44; N, 11.93; S, 8.15.

2.3 Synthesis of Zn1:

The same procedure as for **Co1** was followed and 0.99 mmol Zn(CF$_3$SO$_3$)$_2$ was used instead of Co(CF$_3$SO$_3$)$_2$.
Yield: 0.74 g (95 %). Anal. Calc. for $C_{27}H_{27}ZnF_6N_7O_6S_2$: C, 41.10; H, 3.45; N, 12.43; S, 8.13. Found: C, 41.33; H, 3.40; N, 12.43; S, 8.12.

2.4 Synthesis of Co2:

A 5 mL tetrahydrofuran (THF) solution of **Co1** (203 mg, 0.26 mmol) in a 50 mL Schlenk tube was purged with dioxygen for approximately 3 minutes at 25°C via a calibrated gas line (flow rate: 15 mL/min). The reaction mixture was stirred vigorously overnight, during which the solution colour changed from pale green to blue-purple, indicating oxidation. The crude mixture was filtered through a 0.45 µm PTFE syringe

filter to remove any particulates, and the filtrate was stored undisturbed at 5°C for several days, yielding bluish crystalline solid of **Co2**.

Yield: 151 mg (81 %) Anal. Calc. for $C_{53}H_{55}Co_2F_9N_{14}O_{10}S_3$: C, 44.42; H, 3.87; N, 13.68; S, 6.71. Found: C, 44.84; H, 3.66; N, 13.54; S, 6.50.

2.5 Synthesis of Zn2:

The same procedure as for **Co2** was followed and 0.26 mmol of **Zn1** was used instead of **Co1**.

Yield: 143 mg (76 %) Anal. Calc. for $C_{53}H_{55}Zn_2F_9N_{14}O_{10}S_3$: C, 44.02; H, 3.83; N, 13.56; S, 6.65. Found: C, 44.24; H, 3.76; N, 13.51; S, 6.22.

2.6 Synthesis of Co3:

A 5 mL solution of **Co1** (203 mg, 0.26 mmol) in dichloromethane (DCM) was prepared in a 50 mL Schlenk tube and cooled to −90 °C using an ethanol/liquid nitrogen bath. The solution was purged with dioxygen for approximately 3 minutes, after that the reaction mixture was stirred vigorously while maintaining the temperature at −90 °C for over 2 hours, during which the color of the solution changed from pale green to bright yellow, indicating oxidation. Unreacted dioxygen was removed by purging the reaction mixture with nitrogen gas for 5 minutes. The mixture was then frozen using a liquid nitrogen bath, and the headspace was evacuated under vacuum. Upon thawing the reaction mixture back to −90 °C, the headspace was charged with nitric oxide gas under stirring. An immediate color change to forest green was observed, consistent with the formation of the peroxynitrite species, **Co1-O_2NO^-**. The reaction mixture was subsequently allowed to warm to room temperature overnight with continuous stirring. The crude product was dried under reduced pressure, redissolved in tetrahydrofuran (THF), and filtered through a 0.45 µm PTFE filter siring. The resulting solution was stored undisturbed at 5 °C for several days, during which time bluish crystalline **Co2** kept precipitating and was collected (~62%) by periodic filtration until no further solid formed. The remaining solution was then dried in vacuo, and the residue was recrystallized from acetonitrile to afford crystalline **Co3**.

Yield: 17 mg (21 %) Anal. Calc. for $C_{53}H_{55}Zn_2F_9N_{14}O_{10}S_3$: C, 44.02; H, 3.83; N, 13.56; S, 6.65. Found: C, 44.24; H, 3.76; N, 13.51; S, 6.22.

2.7 External substrate (anisole) nitration with Co1-O_2NO^-:

In a 25ml Schlenk tube, a mixture of 5mM **Co1** and 90mM anisole in 2 ml DCM is taken and cooled to −90 °C using an ethanol/liquid nitrogen bath. Then the same procedure as in the section of 2.6 is followed to form the peroxynitrate species, **Co1-O_2NO^-**. After allowing the reaction mixture to warm up to room temperature overnight with continuous stirring nitromethane (90mM) as an internal standard was added for quantification. The resulted mixture is passed through a silica plug and then 5ml DCM-*d*$_2$ is passed through the silica plug. ^{1}H NMR analysis of the filtrate shows formation of 2-nitroanisole and 4-nitroanisole in 37.2 % yield each with respect to the starting **Co1** used as evident from the marker peaks of the -OMe groups (3.89 ppm and 3.94 ppm for 2-nitroanisole and 4-nitroanisole respectively, see Figure S14). Furthermore, a controlled experiment is also performed in absence of **Co1** where those peaks at 3.89 ppm and 3.94 ppm didn't appear, confirming the aromatic nitration is occurring only by peroxynitrate species, **Co1-O_2NO^-**.

3 NMR spectra of L:

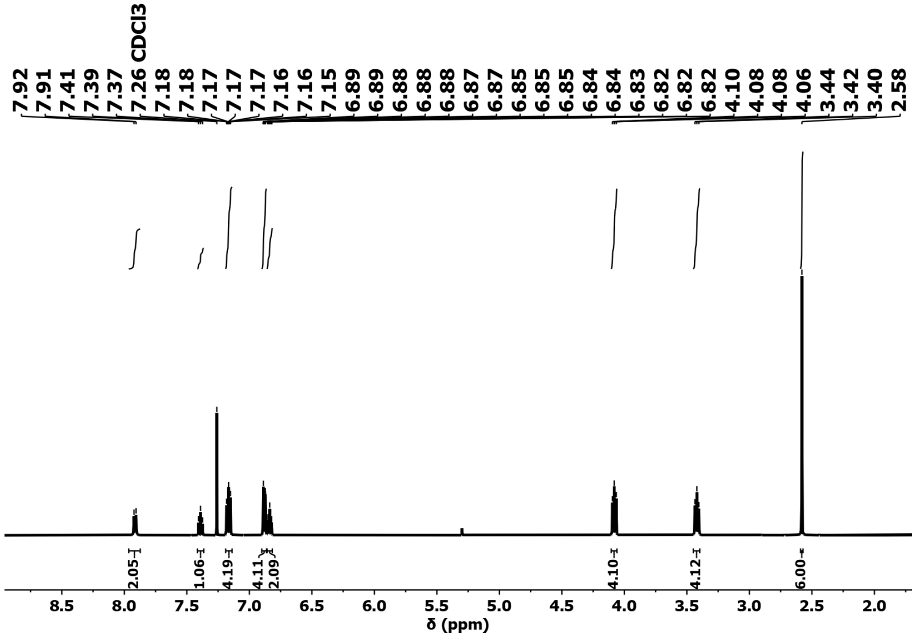

Figure S1. ^{1}H NMR of ligand (**L**).

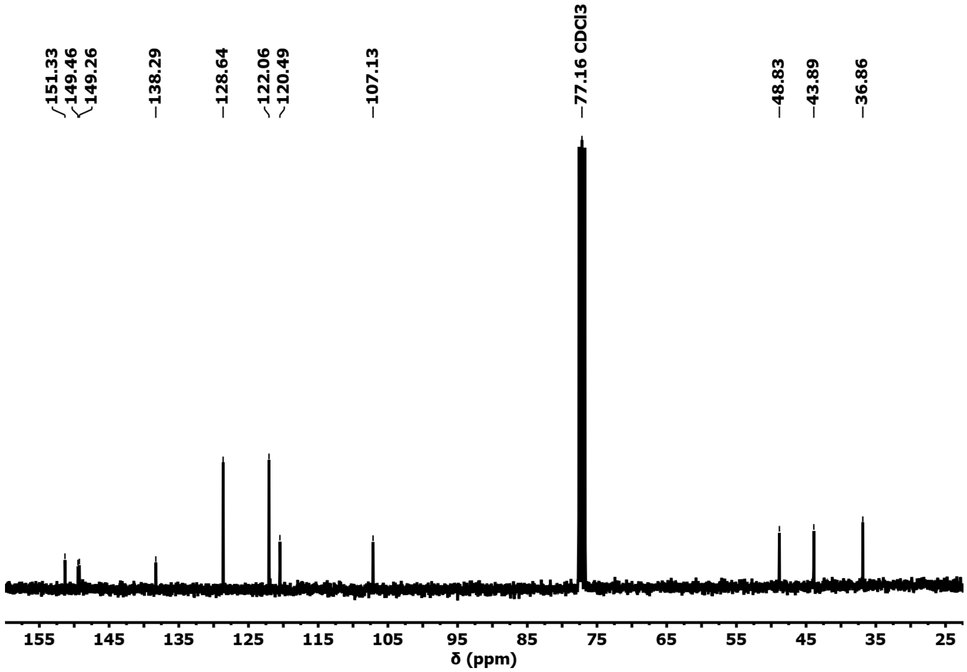

Figure S2. ^{13}C NMR of ligand (^{NNN}**L**).

4 Cyclic Voltammetry:

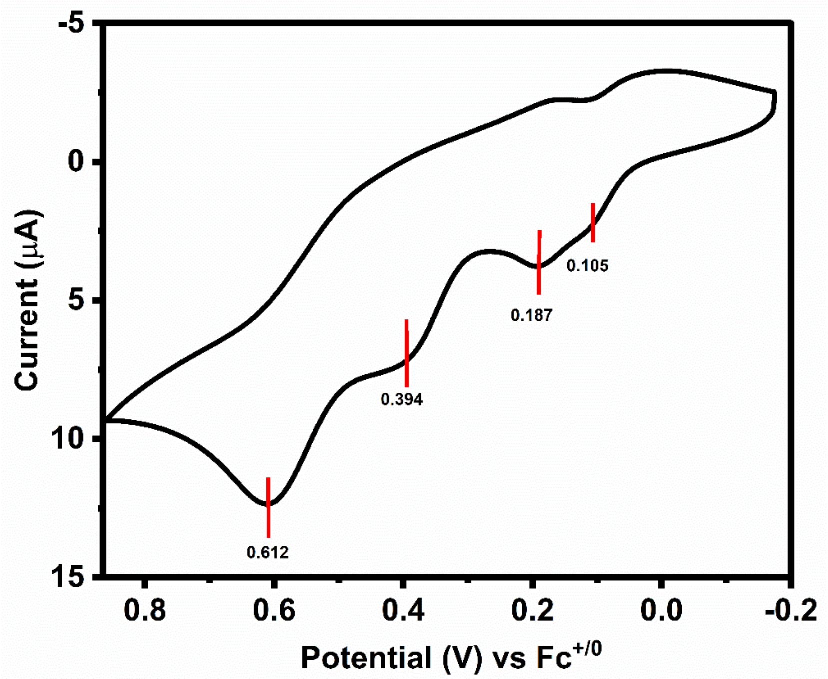

Figure S3. Cyclic voltammetry traces of complexes **Co1**.

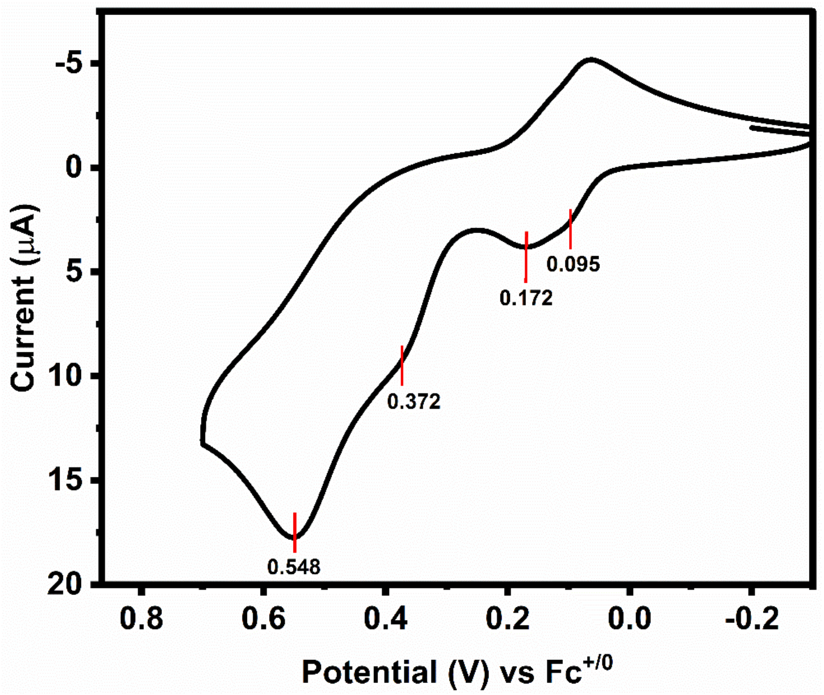

Figure S4. Cyclic voltammetry traces of complexes **Zn1**.

5 NMR spectra of Zn2:

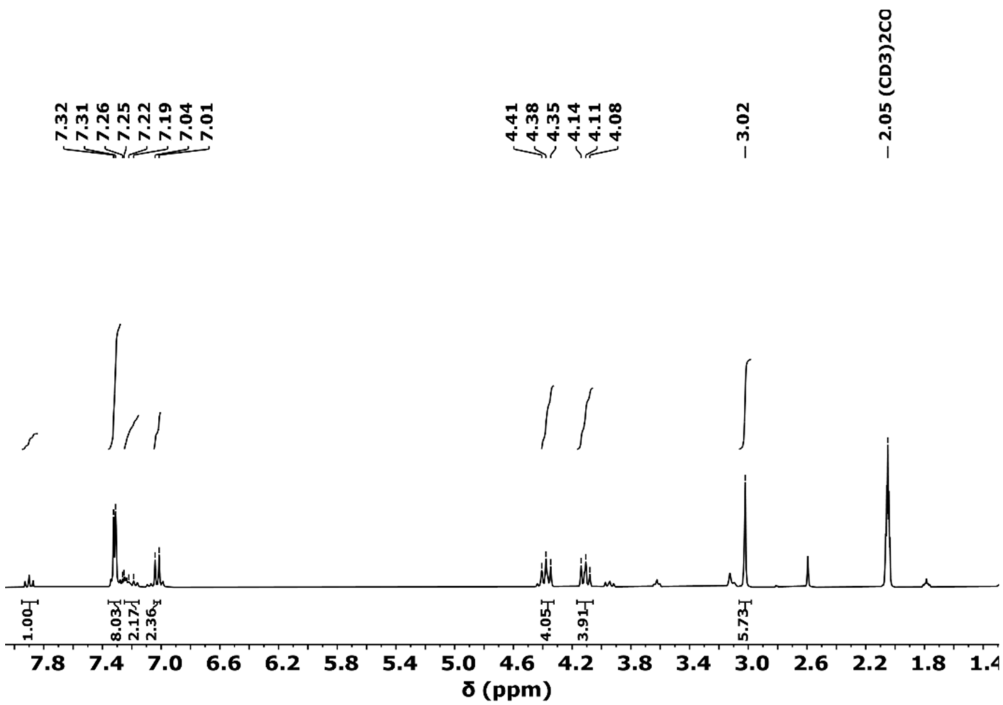

Figure S5. ^{1}H NMR of **Zn2**.

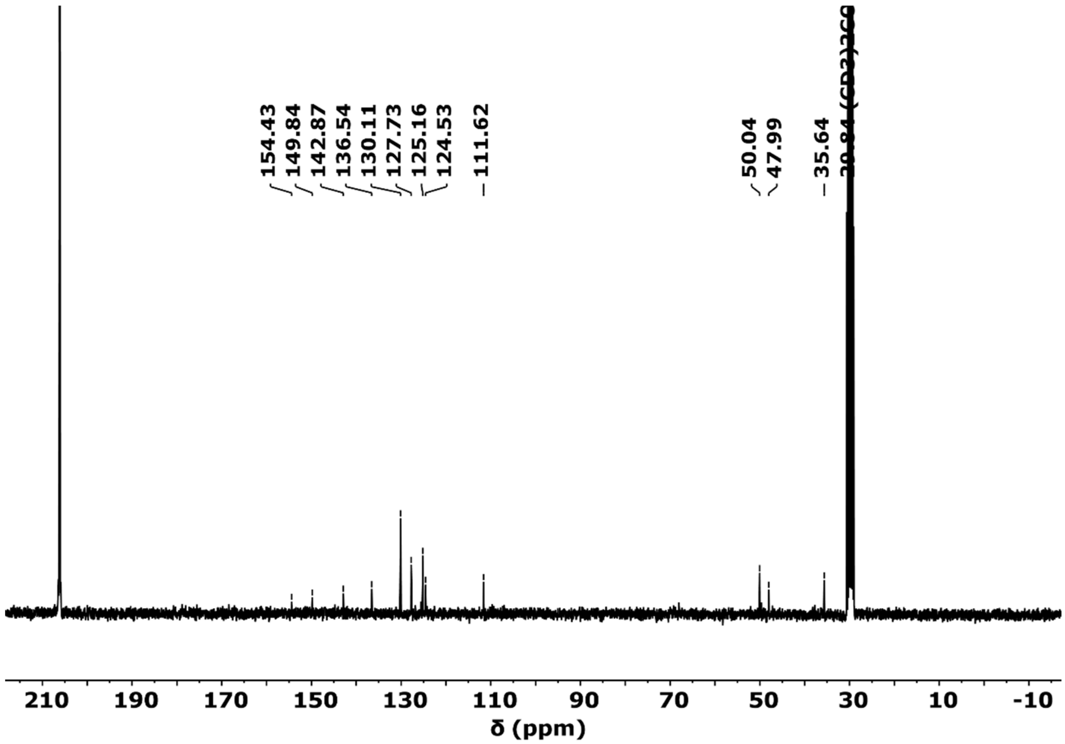

Figure S6. ^{13}C NMR of **Zn2**.

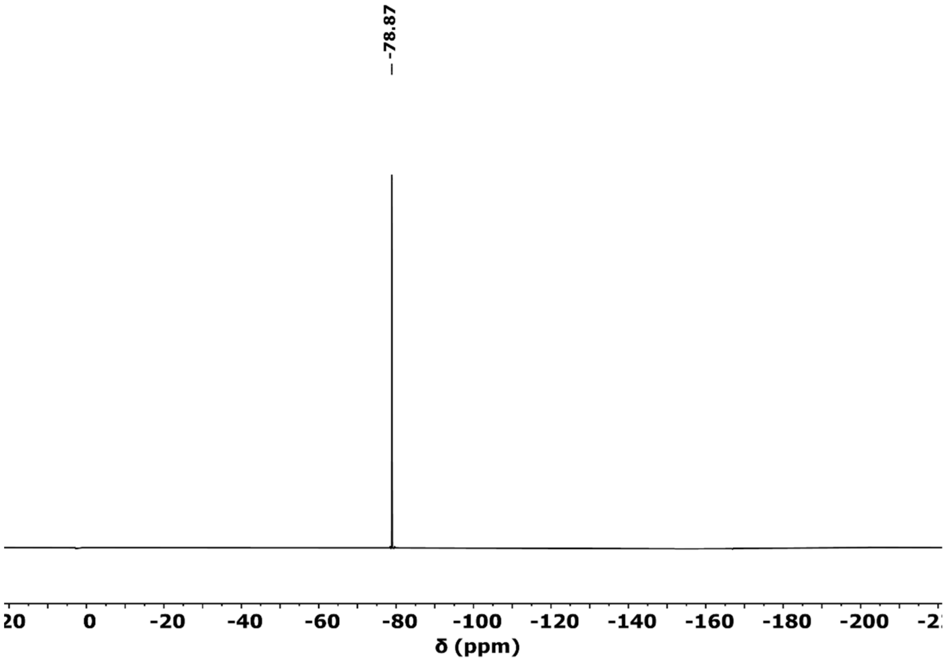

Figure S7. ^{19}F NMR of **Zn2**.

6 SQUID magnetometry of Co2:

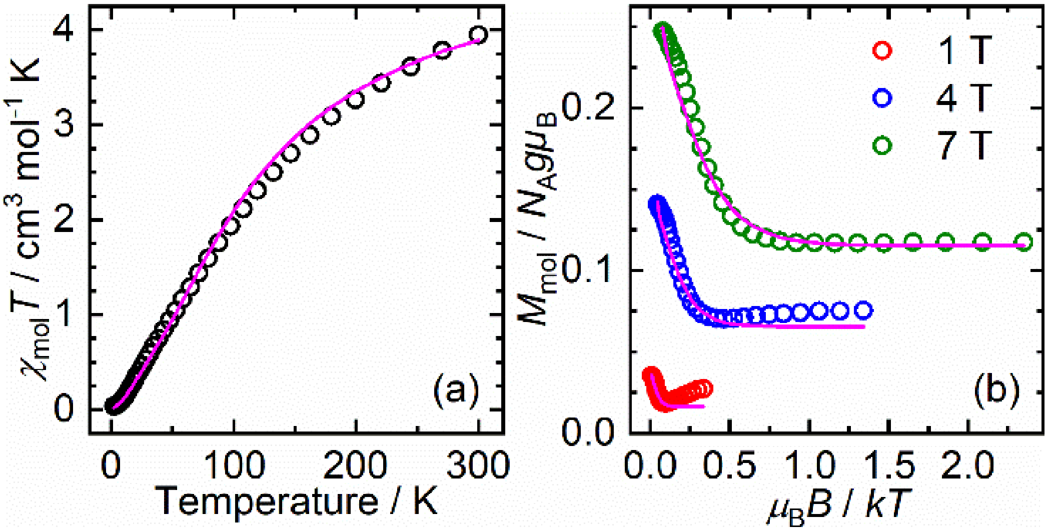

Figure S8. Data points and simulated curves (magenta) for SQUID magnetic measurements on powder samples of **Co2**: (a) χT vs T recorded at B_0 = 0.1 T and (b) iso-field magnetization M_{mol} recorded at 1 (red), 4 (blue), and 7 (green) T vs $\mu_B B_0/kT$. In (a), the χT value close to 0 at 2 K readily indicates antiferromagnetic coupling of the two cobalt(II) sites. The early onset of the rise of χT with increasing temperature shows the interaction to be comparatively weak. To evaluate the exchange coupling constant J, global simulations of the susceptibility and VFVT magnetization data were performed employing the spin Hamiltonian in eq. S2. A reasonably simple model was chosen to avoid over parametrization, with axial $\boldsymbol{g}$ tensors, and identical and collinear (due to the lack of information on their molecular orientation) $\boldsymbol{g}$ and $\boldsymbol{D}$ tensors for both sites. The optimized spin Hamiltonian parameters obtained from least-squares fitting are $g_\perp$ = 2.50, $g_\parallel$ = 1.91, $\boldsymbol{D}$ = [24.6, –2.6, –22.0] cm^{-1} and J = –17.4 cm^{-1}. The $\boldsymbol{D}$ tensor corresponds to D = 36.9 cm^{-1} and E = 9.7 cm^{-1} (E/D = 0.26) according to the Blumberg convention[5] ([D_z'| > [D_y'| > [D_x'|, D = 3D_z'/2, E = (D_x'- D_y')/2), with the corresponding rotation of the $\boldsymbol{D'}$ eigenframe by Euler angles [α, β, γ] = [0, 90, 90]°. Despite the deviations of the simulated from the experimental VFVT magnetization data, which are thought to arise from the assumed simplifications, the characteristic curvature of the χT curve at low temperatures nevertheless allows for a reliable determination of the J value.

7 rRaman spectra of Co1-$O_2^{\bullet -}$:

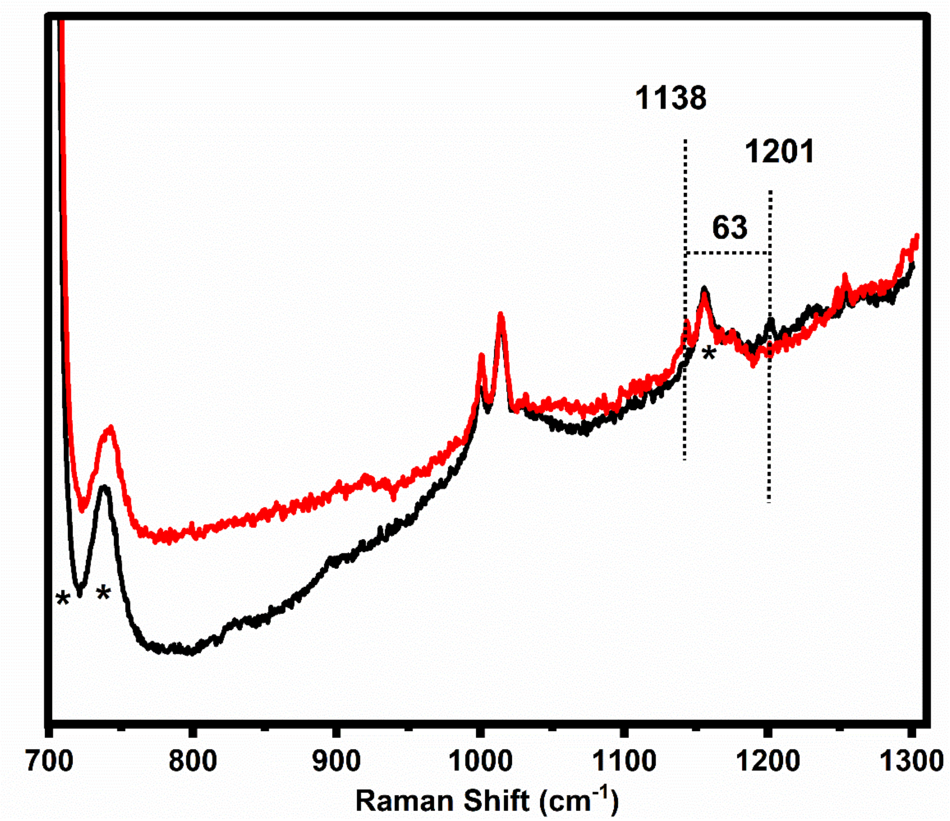

Figure S9. Raw data as collected from rRaman measurements of **Co1-$^{16}O_2^{\bullet -}$**(black) and **Co1-$^{18}O_2^{\bullet -}$**(red) in CH_2Cl_2 at –90 °C (λ_{exc} = 407 nm).

8 DFT computations for Co1-$O_2^{\bullet-}$:

The starting structures for the geometry optimizations all employed the LCo framework in the geometry as found in the XRD structure of **Co1**, with the superoxide ligand coordinated to the Co center in varying end-on and side-on binding geometries and with or without a triflate ion additionally bound to the Co atom. Geometry optimizations were carried out for broken-symmetry (BS) total spin states $S_t = 3/2$ ($S_1 = 2$, $S_2 = 1/2$) and $S_t = 1/2$ ($S_1 = 3/2$, $S_2 = 1$), as well as without using the BS formalism for spin states $S = 5/2$, $S = 3/2$ and $S = 1/2$. Since for the stable side-on superoxo configuration, the triflate ion, if present, was found to dissociate from the Co center during geometry optimization, we focused in our further analysis on the superoxo structures obtained in the absence of triflate.

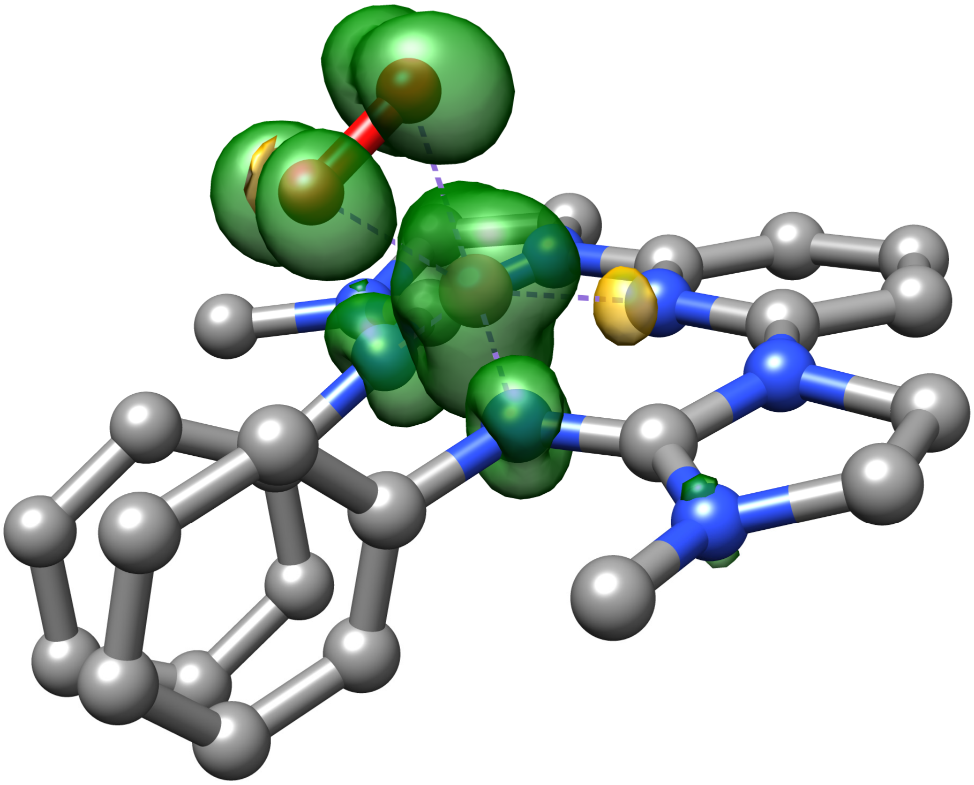

Figure S10. Calculated spin-density map (green: positive (α) density, yellow: negative (β) density, isovalue 0.004 a.u.) on the lowest-energy $S_t = 3/2$ model for **Co1-$O_2^{\bullet-}$**. Hydrogen atoms are omitted for clarity.

Table S1. Cartesian coordinates of the lowest-energy S_t = 3/2 model for **Co1-O$_2^{\bullet-}$** (Figure 7) in Å.

O	8.80697	5.49593	7.77624	C	7.68785	8.88517	2.37074
O	8.44115	5.55267	6.53436	C	7.1009	7.93377	3.1982
N	9.18488	8.29203	6.24952	C	7.57352	7.73703	4.48699
N	10.06237	9.21284	8.235	Co	9.98563	6.61578	6.67538
N	11.45601	7.40216	7.57212	H	11.27829	7.21443	3.24259
N	12.92089	5.6932	6.78771	H	10.12992	6.89453	1.06909
N	11.03136	5.71649	5.38275	H	8.56785	4.99703	0.76694
N	13.02137	4.47882	4.92366	H	8.15462	3.42888	2.6377
N	8.37574	10.29079	7.25384	H	9.32177	3.74377	4.79749
C	9.19206	9.23961	7.16169	H	14.57804	6.64439	8.58343
C	11.25127	8.50949	8.33505	H	14.14882	8.56225	10.04976
C	12.65476	6.76482	7.6204	H	12.03196	9.80163	9.86649
C	12.25448	5.30865	5.63658	H	10.09559	10.00447	4.48274
C	10.37762	5.48832	4.13324	H	9.22854	10.37322	2.19247
C	10.60119	6.38472	3.09283	H	7.31552	9.0327	1.3655
C	9.95497	6.20012	1.87972	H	6.26922	7.3418	2.83957
C	9.07606	5.13586	1.71221	H	7.10675	7.01078	5.13618
C	8.84086	4.25608	2.76388	H	15.07865	4.05701	5.0633
C	9.48745	4.4306	3.97919	H	14.02663	3.04517	6.07452
C	14.19193	4.05547	5.69329	H	14.46969	4.63435	7.77445
C	14.26823	5.08479	6.80649	H	15.01359	5.85147	6.59469
C	12.64958	3.66489	3.76953	H	11.94398	2.88299	4.05183
C	13.63899	7.16429	8.52247	H	13.5643	3.20425	3.40437
C	13.39481	8.24441	9.34253	H	12.22758	4.27278	2.97776
C	12.20745	8.94019	9.24717	H	10.67315	11.1275	8.86757
C	9.86056	10.42594	9.05643	H	9.83225	10.17189	10.11257
C	8.53415	10.96473	8.5449	H	7.7002	10.69264	9.19547
C	7.12014	10.46635	6.52804	H	8.54716	12.04442	8.41392
C	8.64332	8.49726	4.9486	H	7.29765	10.65316	5.47312
C	9.24948	9.43726	4.11693	H	6.62168	11.33064	6.95832
C	8.76158	9.63712	2.83388	H	6.48121	9.59048	6.64291

9 EPR spectrum of the observed $^{\bullet}NO_2$ radical:

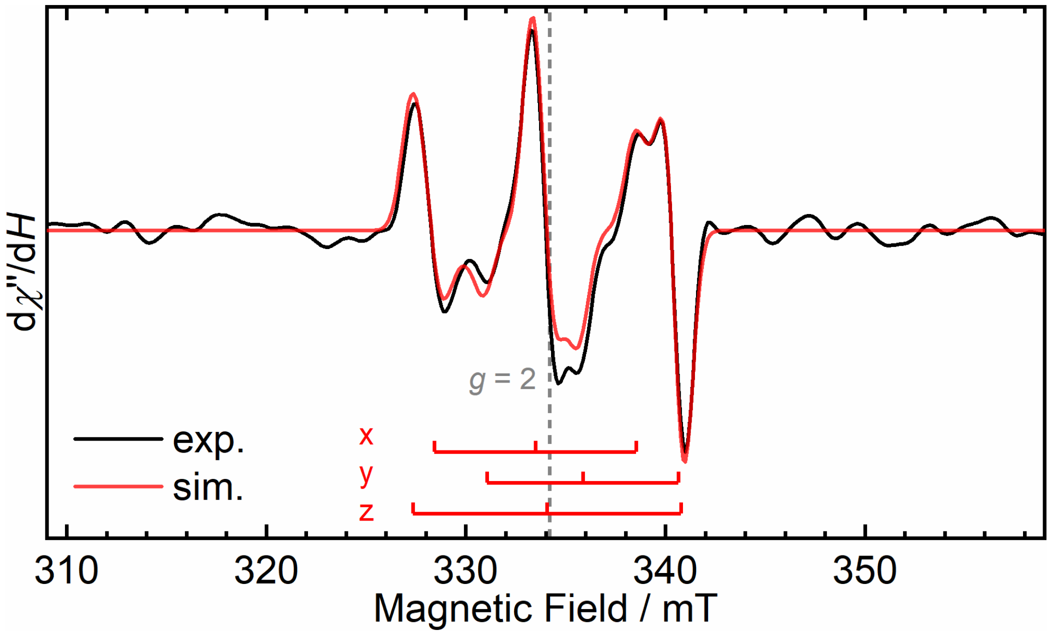

Figure S11. Section of the X-band EPR spectrum in main text Figure 11B for the thermal decomposition of **Co1-O$_2$NO$^-$** in DCM (1 mM), magnifying the g = 2 region with the $^{\bullet}NO_2$ radical signal. Experimental and simulated spectra are shown in black and red, respectively. The splitting pattern for the principal axes directions is depicted on the bottom. The optimized simulations parameters are $\boldsymbol{g}$ = [2.0043, 1.9901, 2.0008] and $\boldsymbol{A}$ = [142, 134, 188] MHz, which is in excellent agreement with the values found for $^{\bullet}NO_2$ in various solid environments ($\boldsymbol{g}$ = [2.005–2.006, 1.991–1.993, 2.000–2.003] and $\boldsymbol{A}$ = [136–149, 128–141, 173–198] MHz).[25] Experimental conditions: see Figure 11.

10 Peroxynitrite decay kinetic studies:

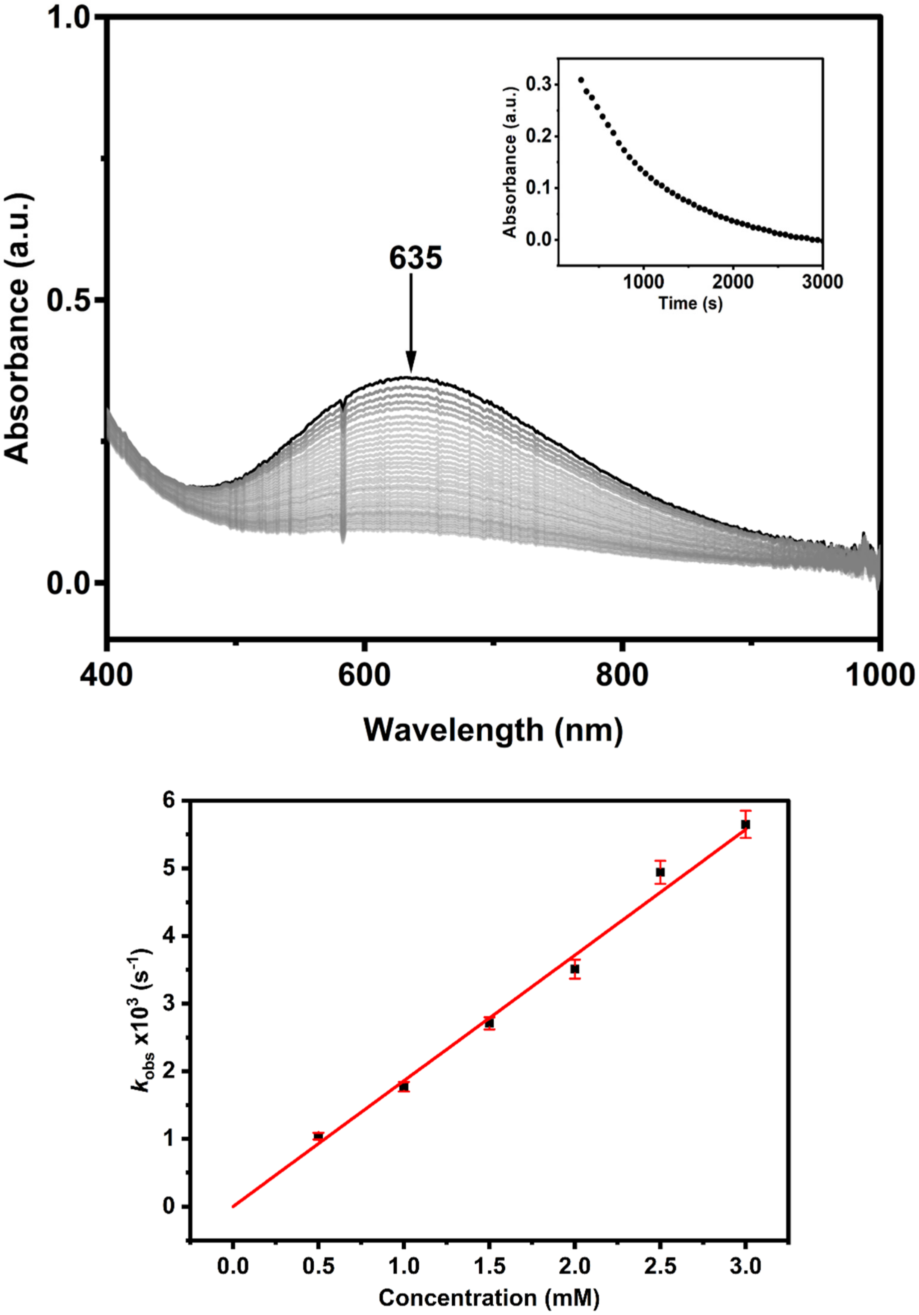

Figure S12. (Up) UV-Vis spectral changes as observed while decaying **Co1-O_2NO^-** at room temperature. Inset describes the change of absorbance at 635nm with respect to time. (Down) Kinetic studies (slope = 1.86) carried out for self-decay of **Co1-O_2NO^-** as a function of increasing concentration at room temperature.

11 Dioxygen and •NO_2 detection by GC-Gas:

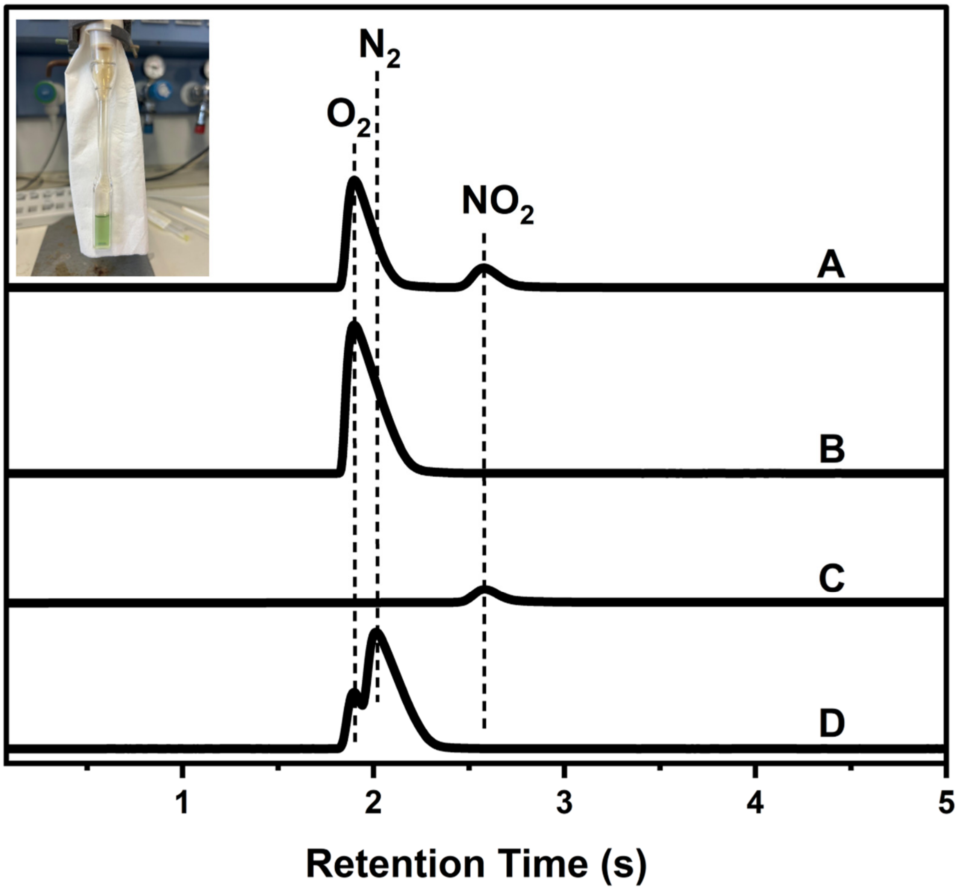

Figure S13. (A) GC-gas analysis from the reaction headspace after decaying **Co1-O_2NO^-** at room temperature, (B) Authentic O_2 sample, (C) Authentic •NO_2 sample, (D) Atmospheric Gas Mixture. Inset: Picture of the reaction cuvette as observed after **Co1-O_2NO^-** at room temperature. Experimental condition: see SI experimental section **2.7**.

12 External Substrate nitration reactivity:

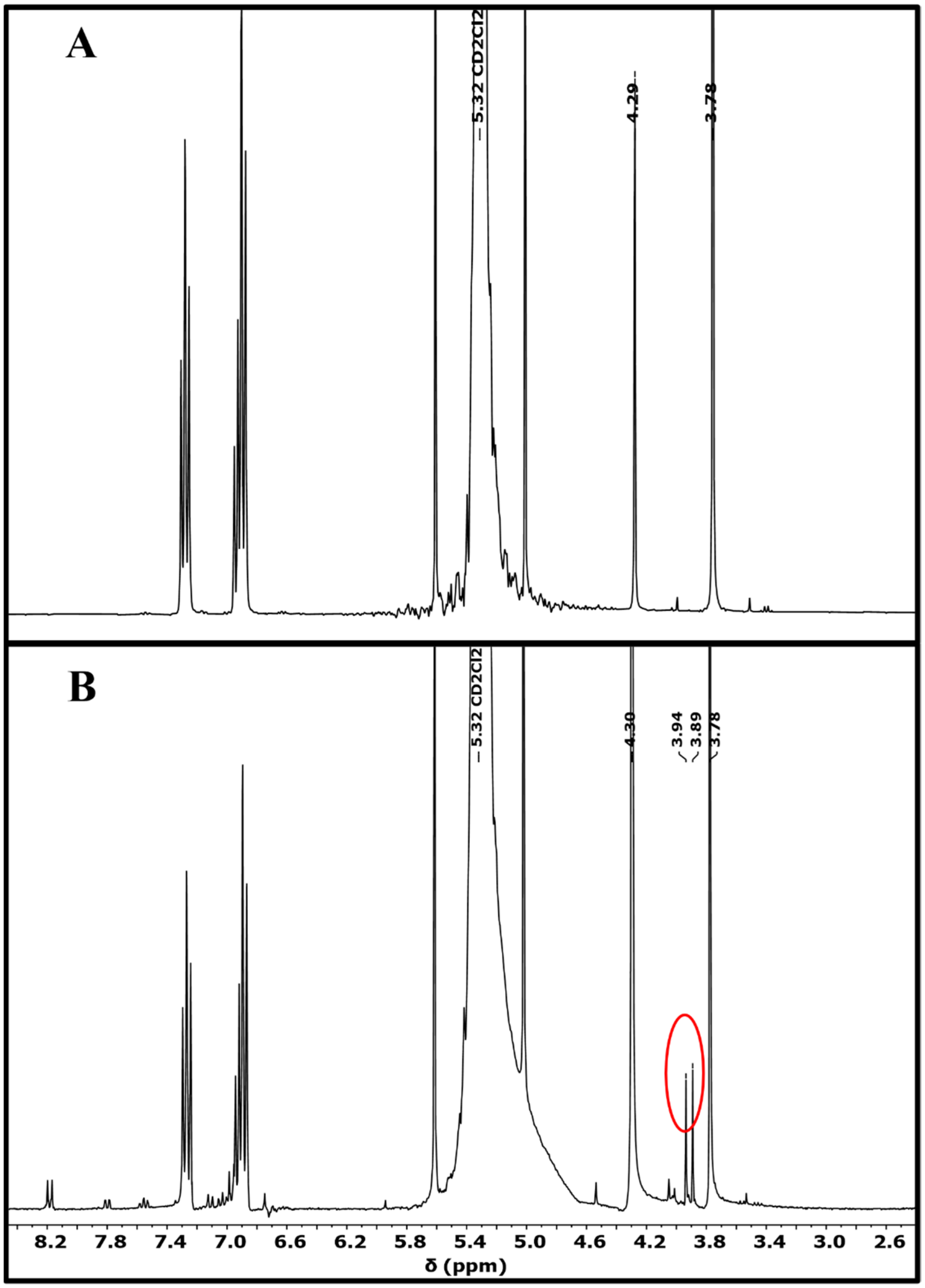

Figure S14. (A) Control experiment. (B) Detection of *ortho* and *para*-nitrated products of anisole in ^{1}H NMR. Marker Peaks: (–OMe group) Anisole = 3.78 ppm; (–OMe group) 2-nitroanisole = 3.89 ppm; (–OMe group) 4-nitroanisole = 3.94 ppm; (–Me group) nitromethane (internal standard) = 4.30 ppm. Conversion with respect to Co_1-O_2NO (catalyst) is 37.2% (each). Experimental condition: see SI experimental section **2.7**.

13 Table S2. XRD determined selected bond lengths.

complex	Fe-N$_{ligand-I}$		Fe-O$_{ligand-II}$	
	atoms	distances	atoms	distances
Co1	Co1-N1	2.005(3)	Co1-O$_{hydroxo}$	1.979(2)
	Co1-N2	1.930(3)		
	Co1-N3	1.955(3)		
Co2	Co1-N1	2.017(2)	Co1-O$_{hydroxo}$	1.900(2)
	Co1-N2	1.958(2)		
	Co1-N3	1.937(2)		
	Co2-N1	2.004(2)	Co2-O$_{hydroxo}$	1.899(2)
	Co2-N2	1.956(2)		
	Co2-N3	1.954(2)		
Zn2	Zn1-N1	2.012(2)	Zn1-O$_{hydroxo}$	1.914(2)
	Zn1-N2	1.957(3)		
	Zn1-N3	1.961(2)		
	Zn2-N1	2.024(3)	Zn2-O$_{hydroxo}$	1.903(2)
	Zn2-N2	1.962(3)		
	Zn2-N3	1.985(3)		
Co3	Co1-N1	2.073(15)	Co1-O$_{hydroxo}$	2.189(10)
	Co1-N2	2.114(13)	Co1-N$_{nitrate}$	2.142(11)
	Co1-N3	2.064(14)	Co1-N$_{nitrate}$	2.179(10)

14 Table S3. EXAFS simulation parameters:[a]

shell	Co-N	Co-N/O	Co-C	Co-O	R_F
**Co1 (fit 1)**					
N	3*	2.0	6*	0.1	
R	2.01	2.14	2.95	2.87	8.1
$2\sigma^2$ x10^3	10	2*	76	2*	
**Co1 (fit 2)**					
N	3*	1*	6*	0.1	
R	2.10	1.95	2.91	2.80	15.5
$2\sigma^2$ x10^3	4	<1	51	2*	
**Co1-$O_2^{\bullet -}$ (fit 1)**					
N	3*	2.1	6*	0.3	
R	1.98	2.13	2.93	2.75	8.0
$2\sigma^2$ x10^3	15	2*	86	2*	
**Co1-$O_2^{\bullet -}$ (fit 2)**					
N	3*	1*	6*	0.4	
R	2.09	1.92	2.95	2.74	16.7
$2\sigma^2$ x10^3	5	<1	65	2*	
**Co1-O_2NO (fit 1)**					
N	3*	0.8	6*	0.6	
R	1.91	2.06	2.82	2.76	9.2
$2\sigma^2$ x10^3	8	2*	130	2*	
**Co1-O_2NO (fit 2)**					
N	3*	0.9	6*	0.6	
R	1.98	1.88	2.84	2.77	8.8

$2\sigma^2$ x10^3	16	2*	189	2*	
**Co1-O_2NO (fit 3)**					
N	3*	0.6	6*	0.7	
R	1.93	1.79	2.72	2.43	5.6
$2\sigma^2$ x10^3	19	2*	50	2*	

[a]Coordination number, N [per Co atom]; interatomic distance, R [Å]; Debye-Waller factor, $2\sigma^2$ x10^3 [$Å^2$]; fit error sum, R_F, calculated for a reduced distance range of 1-3 Å [%]. Alternative fit results are shown for each complex, the parameters of which (i.e., the overall ca. 0.08 Å longer Co-N/O bond lengths compared to the XRD structure of Co1 with a 4-coordinated metal, unphysically small Debye-Waller factor for a 4-coordinate site, and 2-fold worse fit quality) suggest at least 5-coordinate Co(II) sites in Co1 and Co1-$O_2^{\bullet -}$ (fit 2) in acetone solution, possibly due to solvent binding at the metal. Co1-O_2NO, all fit results (i.e., the shorter Co-N/O bonds) and in particular fit 3 suggested a 4-coordinate Co site with possibly an O_2-containing ligand with its 1st O-atom at ca. 1.80-1.90 Å and its 2nd O-atom around 2.45-2.70 Å distance to the metal. EXAFS simulation curves in Fig. 6 correspond to fits 1, 1, and 3 for the three complexes.

15 Table S4. Crystallographic data:

Complex	Co1	Co2	Zn2	Co3
Empirical formula	$C_{27}H_{27}CoF_6N_7O_6S_2$	$C_{69}H_{85}Co_2F_9N_{14}O_{14}S_3$	$C_{54}H_{59}F_9N_{14}O_{11}S_3Zn_2$	$C_{26}H_{25}CoF_3N_{10}O_{10}S$
Formula weight	782.61	1719.55	1478.11	785.55
Temperature (K)	100	100	100	100
Wavelength (Å)	0.71073	0.71073	0.71073	0.71073
space group	$P2_1/c$	$P2_1/n$	$P2_1/n$	$P2_1/c$
Crystal size(mm)	0.45x0.2x0.09	0.22x0.14x0.07	0.20x0.19x0.07	0.10x0.08x0.04
a (Å)	27.244(4)	14.269(1)	20.219(1)	13.412(9)
b (Å)	12.407(2)	14.449(1)	15.409(1)	14.635(10)
c (Å)	18.782(3)	36.986(3)	21.990(1)	16.006(10)
α (°)	90	90	90	90
β (°)	96.803(5)	95.509(2)	112.339(2)	91.869(17)
γ (°)	90	90	90	90
Volume/$Å^3$	6303.9(17)	7590.0(10)	6337.1(5)	3140.0(4)
Z	8	4	4	4
ρ_{calc}(g/cm^3)	1.649	1.505	1.549	1.662
F(000)	3192	3568	3032	1604.0
μ/mm^{-1}	0.767	0.614	0.951	0.705
Nref	15897	15642	11678	3697
Goodness-of-fit (GooF)	1.150	1.023	1.035	1.092
Final R indexes [all data]	R_1 = 0.0634 wR_2 = 0.1290	R_1 = 0.0463 wR_2 = 0.1295	R_1 = 0.0472 wR_2 = 0.1248	R_1 = 0.1003 wR_2 = 0.2936
CCDC No.	2441554	2441553	2441552	2441551

16 References:

(1) Merchán F., Garín J., Martínez V., Meléndez E., Synthesis of 2-Aryliminoimidazolidines and 2-Arylaminobenzimidazoles from Methyl N-Aryldithiocarbamates. Synthesis 1982, 6, 482-484.
(2) Fulmer G. R., Miller A. J. M., Sherden N. H., Gottlieb H. E., Nudelman A., Stoltz B. M., Bercaw J. E., Goldberg K. I., NMR Chemical Shifts of Trace Impurities: Common Laboratory Solvents, Organics, and Gases in Deuterated Solvents Relevant to the Organometallic Chemist. Organometallics 2010, 29(9), 2176–2179.
(3) Bain, G. A.; Berry, J. F., Diamagnetic Corrections and Pascal's Constants. J. Chem. Educ. 2008, 85 (4), 532.
(4) Stoll, S.; Schweiger, A., EasySpin, a comprehensive software package for spectral simulation and analysis in EPR. J. Magn. Reson. 2006, 178 (1), 42-55.
(5) Blumberg, W. E., The EPR of High Spin Fe3+ in Rhombic Fields. 1967, 119-133.
(6) Neese, F., The ORCA program system. WIREs Comput. Molec. Sci. 2011, 2 (1), 73-78.
(7) Neese, F., Software update: The ORCA program system—Version 5.0. WIREs Comput. Molec. Sci. 2022, 12 (5).
(8) Neese, F., The SHARK integral generation and digestion system. J. Comput. Chem. 2022, 44 (3), 381-396.
(9) Neese, F.; Wennmohs, F.; Becker, U.; Riplinger, C., The ORCA quantum chemistry program package. J. Chem. Phys. 2020, 152 (22).
(10) van Lenthe, E.; Baerends, E. J.; Snijders, J. G., Relativistic regular two-component Hamiltonians. J. Chem. Phys. 1993, 99 (6), 4597-4610.
(11) van Lenthe, E.; Baerends, E. J.; Snijders, J. G., Relativistic total energy using regular approximations. J. Chem. Phys. 1994, 101 (11), 9783-9792.
(12) van Wüllen, C., Molecular density functional calculations in the regular relativistic approximation: Method, application to coinage metal diatomics, hydrides, fluorides and chlorides, and comparison with first-order relativistic calculations. J. Chem. Phys. 1998, 109 (2), 392-399.
(13) Pantazis, D. A.; Chen, X.-Y.; Landis, C. R.; Neese, F., All-Electron Scalar Relativistic Basis Sets for Third-Row Transition Metal Atoms. J. Chem. Theory Comput. 2008, 4 (6), 908-919.
(14) Barone, V.; Cossi, M., Quantum Calculation of Molecular Energies and Energy Gradients in Solution by a Conductor Solvent Model. J. Phys. Chem. A 1998, 102 (11), 1995-2001.
(15) Grimme, S.; Antony, J.; Ehrlich, S.; Krieg, H., A consistent and accurate ab initio parametrization of density functional dispersion correction (DFT-D) for the 94 elements H-Pu. J. Chem. Phys. 2010, 132 (15), 154104.
(16) Becke, A. D., Density-functional exchange-energy approximation with correct asymptotic behavior. Phys. Rev. A 1988, 38 (6), 3098-3100.
(17) Becke, A. D., Density‐functional thermochemistry. III. The role of exact exchange. J. Chem. Phys. 1993, 98 (7), 5648-5652.
(18) Lee, C.; Yang, W.; Parr, R. G., Development of the Colle-Salvetti correlation-energy formula into a functional of the electron density. Phys. Rev. B 1988, 37 (2), 785-789.
(19) Neese, F.; Olbrich, G., Efficient use of the resolution of the identity approximation in time-dependent density functional calculations with hybrid density functionals. Chem. Phys. Lett. 2002, 362 (1-2), 170-178.
(20) Neese, F.; Wennmohs, F.; Hansen, A.; Becker, U., Efficient, approximate and parallel Hartree–Fock and hybrid DFT calculations. A ‘chain-of-spheres’ algorithm for the Hartree–Fock exchange. Chem. Phys. 2009, 356 (1-3), 98-109.
(21) Weigend, F., Accurate Coulomb-fitting basis sets for H to Rn. Phys. Chem. Chem. Phys. 2006, 8 (9), 1057.
(22) Perdew, J. P., Density-functional approximation for the correlation energy of the inhomogeneous electron gas. Phys. Rev. B 1986, 33 (12), 8822-8824.
(23) Neese, F., An improvement of the resolution of the identity approximation for the formation of the Coulomb matrix. J. Comput. Chem. 2003, 24 (14), 1740-1747.
(24) Pettersen, E. F.; Goddard, T. D.; Huang, C. C.; Couch, G. S.; Greenblatt, D. M.; Meng, E. C.; Ferrin, T. E., UCSF Chimera—A visualization system for exploratory research and analysis. J. Comput. Chem. 2004, 25 (13), 1605-1612.
(25) Lunsford, J. H., EPR spectra of radicals formed when NO_2 is adsorbed on magnesium oxide. *J. Colloid Interface Sci.* **1968,** *26* (3), 355-360.
